# *DEFECTIVE EMBRYO AND MERISTEMS1* (*DEM1*) Is Essential for Cell Proliferation and Cell Differentiation in Tomato

**DOI:** 10.3390/plants11192545

**Published:** 2022-09-28

**Authors:** Louisa Matthew, Melquiades E. C. Reyes, Christopher W. G. Mann, Alasdair W. McDowall, Andrew L. Eamens, Bernard J. Carroll

**Affiliations:** 1 School of Chemistry and Molecular Biosciences, The University of Queensland, St. Lucia, QLD 4072, Australia; 2 Centre for Microscopy and Microanalysis, The University of Queensland, St. Lucia, QLD 4072, Australia; 3 California Institute of Technology, Pasadena, CA 91125, USA; 4 School of Health and Behavioural Sciences, University of the Sunshine Coast, Maroochydore, QLD 4558, Australia

**Keywords:** tomato (*Solanum lycopersicum*), *DEFECTIVE EMBRYO AND MERISTEMS1* (*DEM1*) gene, cell proliferation, cell differentiation, vegetative development, leaf development

## Abstract

Most flowering plant species contain at least two copies of the *DEFECTIVE EMBRYO AND MERISTEMS* (*DEM*) gene with the encoded DEM proteins lacking homology to proteins of known biochemical function. In tomato (*Sl*; *Solanum lycopersicum*), stable mutations in the *SlDEM1* locus result in shoot and root meristem defects with the *dem1* mutant failing to progress past the cotyledon stage of seedling development. Generation of a *Somatic Mutagenesis of DEM1* (*SMD*) transformant line in tomato allowed for the characterization of *SlDEM1* gene function past the seedling stage of vegetative development with *SMD* plants displaying a range of leaf development abnormalities. Further, the sectored or stable *in planta* expression of specific regions of the *SlDEM1* coding sequence also resulted in the generation of tomato transformants that displayed a range of vegetative development defects, which when considered together with the *dem1* mutant seedling and *SMD* transformant line phenotypic data, allowed for the assignment of *SlDEM1* gene function to early embryo development, adaxial epidermis cell development, lateral leaf blade expansion, and mesophyll cell proliferation and differentiation.

## 1. Introduction

The correct formation of a multicellular organism requires highly ordered cell proliferation, together with the targeted withdrawal of individual cells from the cell cycle and their subsequent tightly programmed differentiation into specialized cell types for the development of specific tissues and organs [1,2,3]. In plants, the processes of cell proliferation and cell differentiation are largely spatially separated from one another due to the formation of specialized stem cell populations, termed meristems, located at shoot and root apices [4,5]. In the angiosperms, the shoot apical meristem (SAM) is usually a small dome-like structure that consists of a centrally positioned population of slowly dividing stem cells, with a proportion of this cell population eventually migrating to the peripheral zone of the SAM [6,7]. At the SAM periphery, the relocated stem cells start to divide more rapidly as part of the cellular differentiation process to initiate lateral organ formation [8,9]. Taking leaf development as an example, a cohort of cells positioned on the outer side of the peripheral zone of the SAM, is set aside to form a leaf primordium via this cell cohort adopting a different program of development to the other cell populations of the SAM [7,10,11]. Thus, via driving cell proliferation in a new direction, leaf primordia elaborate an axis of growth away from the meristem and flatten laterally to produce a leaf blade within which the various cell types that comprise a leaf can fully differentiate to adopt their specific adult fates [12,13].

A large collection of transcription factors has been demonstrated to occupy a central role in controlling cell proliferation and cell differentiation as part of leaf development [14,15,16,17,18,19]. One such family of transcription factors are the KNOTTED (KN)-like homeobox domain transcription factors with the *KNOTTED1* (*KN1*) gene of maize (*Zea mays*) being the first identified family member [20,21]. In maize, knot-like outgrowths form on the veins of leaves of the dominant *kn1* mutant, a phenotypic consequence demonstrated to result from excessive rounds of cell division, and which led to the naming of the causative gene, *KN1* [22]. Since the identification of the maize *KN1* gene, numerous other members of the *KN*-like transcription factor gene family have been identified in *Arabidopsis* (*Arabidopsis thaliana*), rice (*Oryza sativa*) and soybean (*Glycine max*) [23,24,25]. Based on sequence similarity and overlapping expression domains, members of the *KN* transcription factor gene family are further divided into two classes, namely class I (*KNOX1*) and class II (*KNOX2*) genes [26,27]. Most *KNOX1* genes are expressed in the SAM and direct roles in meristem initiation and maintenance by promoting cell proliferation and inhibiting cell differentiation [26,28]. In contrast, the primary role of KNOX2 proteins is to antagonize *KNOX1* gene function, with the expressional and functional interplay of *KNOX1* and *KNOX2* genes largely defining developmental patterning in many plant species [29,30,31].

Similar to the KN-like transcription factors, the PHANTASTICA (PHAN) family of MYB domain transcription factors conduct central roles in controlling cell proliferation and cell differentiation in leaf development. The founding member of the *PHAN* transcription factor gene family was first identified in *Antirrhinum majus* (*Antirrhinum*) [32], with orthologs subsequently identified in maize (*ROUGHSHEATH2*) [33], *Arabidopsis* (*ASYMMETRIC LEAVES1*) [34], rice (*OSH3*) [35] and tomato (*SlPHAN*) (*Solanum lycopersicum*) [36]. Via characterization of plant lines harboring mutations in these *PHAN* gene family members, the different degrees of severity of the developmental defects displayed by each respective mutant line, has identified roles for PHAN transcription factors in SAM maintenance, leaf blade development, and the control of dorsoventral asymmetry of leaves [32,33,34,35,36,37]. At the molecular level, and as demonstrated most clearly in *Arabidopsis* [38], PHAN transcription factors appear to primarily mediate their role in leaf development by repressing the expression of specific *KNOX1* genes, a transcriptional change which in turn either promotes or represses cell proliferation and/or cell differentiation in the SAM periphery [36,39,40].

Genome sequence analysis has revealed that dicotyledonous plants such as tomato and *Arabidopsis* encode two distinct copies of the *DEFECTIVE EMBRYO AND MERISTEMS* (*DEM*) gene with the *DEM1* and *DEM2* loci encoding a highly similar protein of unknown biochemical function, except that the encoded proteins interact with RAS-LIKE NUCLEAR PROTEIN (RAN) [41,42]. RAN is conserved in all eukaryotes and plays a fundamental role in nucleus–cytoplasm transport and cell division. Via the use of a modified *Activator*/*Dissociation* (*Ac*/*Ds*) transposon tagging approach, the *DEM1* gene was initially identified in tomato where it was shown to be required for organized cell division in the SAM [41]. More specifically, in tomato, *DEM1* (*SlDEM1*) was shown to be highly expressed in meristematic tissues, namely the SAM and root apical meristem (RAM) and in other differentiating vegetative tissues of tomato [41]. Furthermore, the degree of defective SAM and RAM development in the tomato *dem1* mutant resulted in this plant line failing to proceed past cotyledon emergence as part of the seedling stage of early vegetative development [41]. In contrast to the tomato *dem1* mutant, the *Arabidopsis dem1* and *dem2* single mutants express wild-type-like phenotypes throughout their vegetative development [42]. However, *Arabidopsis* plants carrying a single functional copy of the *DEM1* gene, and which are homozygous for mutation of the *DEM2* gene (specifically, *DEM1/dem1 dem2/dem2* plants), showed defects in meiotic cell division and gamete viability [42]. Repeated attempts to generate an *Arabidopsis dem1 dem2* double mutant via a standard genetic crossing approach have failed, which is consistent with an essential role for DEM proteins in cell division and/or gamete viability [42]. Expression analysis revealed that the *AtDEM2* transcript is more abundant than the *AtDEM1* transcript in all assessed *Arabidopsis* tissues, including the SAM, young and mature leaves, the inflorescence, flowers, floral buds, and siliques [42]. The wild-type appearance of *Arabidopsis dem1* and *dem2* single mutants, together with our failure to generate a *dem1 dem2* double mutant plant, and the overlapping expression of *AtDEM1* and *AtDEM2* suggests that the two encoded *AtDEM* proteins, as well as their tomato orthologs, perform essential, yet partly redundant roles in plant development.

In this study, we show the usefulness of utilizing a transgenic transposon-based approach to assign additional functional roles in plant development to a gene whose dysfunction is lethal in the early stages of development. More specifically, the somatic mutagenesis of the *SlDEM1* locus in the tomato *SMD* transformant line allowed for the identification of the essential requirement of *SlDEM1* gene function in the control of the proliferation and differentiation of specific cell types in tomato leaves. Furthermore, either the sectored or stable expression of transgenes harboring different sections of the *SlDEM1* gene, with or without the regulatory sequences of the tomato *DEM1* gene, not only confirmed the central regulatory role played by the *SlDEM1* gene in specialized aspects of leaf development, but further revealed the absolute requirement of *SlDEM1* in promoting embryo fitness in tomato. Taken together, the results presented here clearly reveal the absolute requirement for *SlDEM1* gene function for normal embryo and vegetative development in tomato. Our findings also provide a solid foundation for the future investigation of the biochemical function of DEM proteins in plant development.

## 2. Results

### 2.1. DEM1 Is Required for Normal Embryogenesis and Meristem Development in Tomato

**Figure 1:**
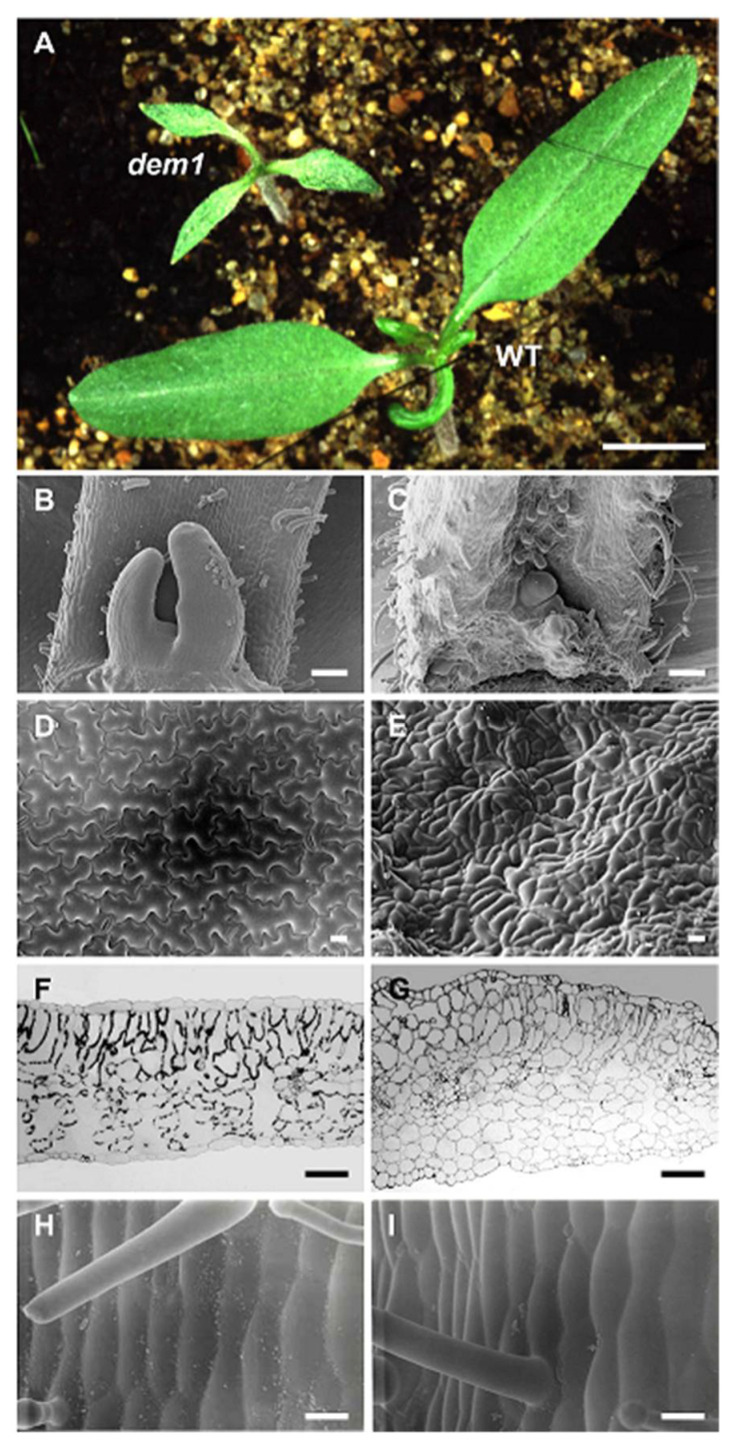
Phenotypes displayed by 2-week-old WT tomato and *dem1* mutant seedlings. (**A**) Phenotypes displayed by 2-week-old WT tomato and *dem1* mutant seedlings. SEM of the shoot apical regions and cotyledons of a 2-week-old WT tomato (**B**) and *dem1* mutant (**C**) seedling post the removal of one cotyledon to facilitate viewing. SEM of the adaxial epidermis of the cotyledons of WT tomato (**D**) and *dem1* mutant (**E**) seedlings. Transverse sections of fully expanded cotyledons of WT tomato (**F**) and *dem1* mutant (**G**) seedlings. SEM of the hypocotyl of WT tomato (**H**) and *dem1* mutant (**I**) seedlings. Scale bars = 5 mm (**A**), 100 μm (**B**,**C**,**F**,**G**), and 30 μm (**D**,**E**,**H**,**I**).

In tomato (*Sl*; *Solanum lycopersicum*), a modified two-element transposon tagging system was initially utilized to introduce a suite of mutations into the *SlDEM1* locus [41,43]. More specifically, the *Activator* (*Ac*) transposon from maize was modified to stabilize its own activity (*sAc*) but to continue to produce the transposase enzyme required for transposition of the *Dissociation* (*Ds*) element. Using this approach, numerous *Ds* insertion events were mapped to the *SlDEM1* locus to generate a suite of tomato *dem1* mutant alleles [41,43]. In direct contrast to 2-week-old wild-type (WT) tomato seedlings, which only produce two cotyledons with a linear shaped blade, *dem1* mutant seedlings of the same age develop two to five cotyledons of greatly reduced overall size and which adopt a highly lanceolate shape (Figure 1A). Furthermore, due to severely defective shoot (SAM) and root apical meristem (RAM) development, the tomato *dem1* mutant fails to progress past the cotyledon stage of seedling development. Scanning electron microscopy (SEM) of the SAM of 2-week-old WT (Figure 1B) and *dem1* seedlings (Figure 1C), clearly revealed that compared to the WT SAM, the cells of the *dem1* SAM were highly disorganized, from which grossly abnormal leaf primordia occasionally formed, but which failed to subsequently differentiate further to form any recognizable ‘true leaf’ structures. SEM further showed the extent to which the *dem1* mutation disrupted cotyledon development with the cells of the adaxial (upper) surface of *dem1* cotyledons being reduced in size and of highly variable shape (Figure 1E), completely lacking the complex and ordered size and shape of the cells of the adaxial epidermis of WT cotyledons (Figure 1D). 

Transverse sections of WT tomato and *dem1* mutant cotyledons not only revealed that *dem1* cotyledons were thicker than WT cotyledons, but that the development of the internal tissues of cotyledons was greatly altered by mutation of the *SlDEM1* locus (Figure 1F,G). Figure 1F shows that the photosynthetically competent palisade mesophyll is composed of a loosely packed row of columnar cells aligned beneath the adaxial epidermis, below which a layer of largely spherical spongy mesophyll cells interspersed with numerous airspaces make up the abaxial (lower) half of the internal tissue of a WT cotyledon. In contrast, the palisade mesophyll of *dem1* cotyledons was revealed to be highly disorganized, being composed of multiple layers of variously sized cells (Figure 1G). In addition, although the spongy mesophyll of the abaxial half of a *dem1* cotyledon is composed of roughly evenly sized cells, the proliferation of spongy mesophyll cells was enhanced. This led to the expansion of spongy mesophyll cells into the adaxial half of *dem1* cotyledons, which in turn, greatly reduced the number of airspaces that formed in the lower half of *dem1* cotyledons. Surprisingly, and despite these phenotypic differences, the abaxial epidermis of *dem1* cotyledons developed normally (data not shown), and as shown in Figure 1H,I SEM images, respectively, WT tomato and *dem1* mutant hypocotyls are phenotypically identical to each other. This indicates that *SlDEM1* gene mutation only impacted specific aspects of embryonic development in tomato. Taken together, the Figure 1 data clearly demonstrates the crucial role played by *SlDEM1* in controlling cell proliferation and cell differentiation for normal SAM and cotyledon development in tomato.

### 2.2. Somatic Mutagenesis of the Tomato DEM1 Gene Identified Further Roles for the DEM1 Protein in Leaf Development

**Figure 2:**
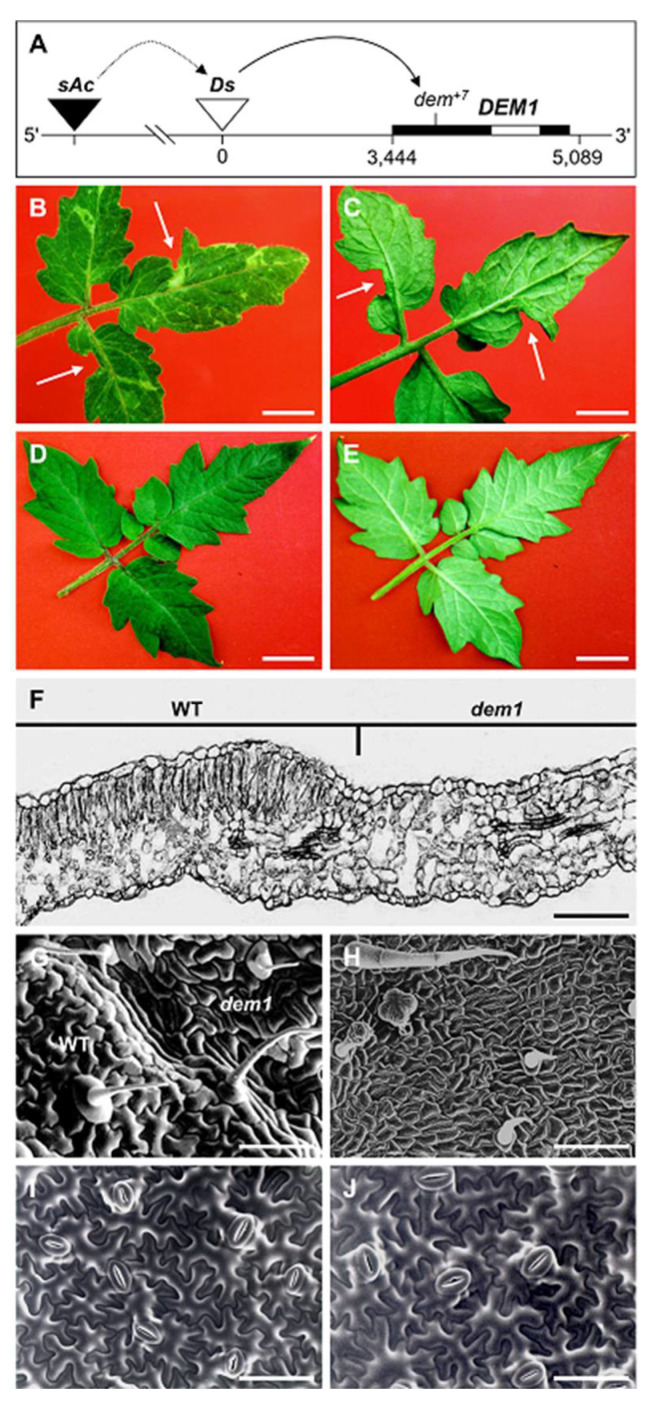
Leaf phenotypes displayed by mature WT tomato plants and the *SMD* transformant line. (**A**) Schematic of the somatic mutagenesis of the *DEM1* locus in the *SMD* transformant line. The stabilized *Ac* (*sAc*; black triangle) produces the transposase which excites *Ds* (white triangle) for its transposition. In the *SMD* transformant line, the 7 bp frameshift insertion into the *DEM1* reading frame (*dem^+7^*) on one heterologous copy of chromosome 4 facilitates the somatic mutagenesis of *DEM1* upon *Ds* transposition into the *DEM1* gene on the second heterologous copy of chromosome 4. (**B**) The adaxial surface of *SMD* leaves show pale green somatic sectors, whereas the abaxial surface of *SMD* leaves (**C**) are phenotypically normal. The white arrows (**B**,**C**) indicate sectors of *SMD* leaves where lateral leaf blade expansion has prematurely terminated. The adaxial (**D**) and abaxial (**E**) surface of leaves of mature WT tomato plants. (**F**) Transverse section through the variegated region (a WT/*dem1* mutant sector boundary) of a mature *SMD* leaf which shows that palisade mesophyll cell fail to form beneath the cells of the adaxial epidermis in *dem1* mutant sectors. (**G**) SEM of the epidermal cells of the adaxial surface of a mature *SMD* leaf across a WT/*dem1* mutant sector boundary. (**H**) SEM of the adaxial surface of a pale green *dem1* mutant leaf sector showing the irregular size and shape of the epidermal cells and the formation of globular trichomes. SEM showing the normal development of the abaxial surface of WT (**I**) and *dem1* mutant (**J**) sectors of a leaf sampled from the *SMD* transformant line. Scale bars = 2.0 cm (**B**–**E**), 100 μm (**F**–**H**), and 50 μm (**I**,**J**).

The seedling lethality of *SlDEM1* gene mutation in tomato led us to develop the *Somatic Mutagenesis of DEM1* (*SMD*) transformant line to attempt to identify additional roles for *SlDEM1* in the subsequent stages of tomato vegetative development. The schematic presented in Figure 2A depicts the genotype of the *SMD* transformant line which at the chromosome level consisted of: (1) a single copy of a 7 base pair (bp) frameshift insertion mutation into the coding sequence of *SlDEM1* (*dem^+7^*) post *Ds* insertion and excision from *SlDEM1*; (2) a proximally located *sAc* transgene on the same homozygous copy of tomato chromosome 4, and; (3) a *Ds* element on the second homologous copy of tomato chromosome 4 which was mapped to a position 3,444 bp upstream of the start codon (ATG) of the *SlDEM1* coding sequence [41]. Due to the tendency of the *Ds* element to transpose locally upon its activation [43,44,45], the *SMD* transformant line was used to further characterize the function of the *DEM1* gene in tomato vegetative development via a *Ds*-directed somatic mutagenesis approach.

The adaxial surface of mature *SMD* leaf blades displayed a variegated pattern of dark green colored somatic sectors where *SlDEM1* was functional (WT sectors) and pale green colored somatic sectors where the *SlDEM1* locus was disrupted by *Ds* transposition (*dem1* mutant sectors) (Figure 2B). The observed phenotype was in direct contrast to that of the adaxial surface of mature WT tomato leaf blades which are a uniform dark green color along the entire length and width of the blade (Figure 2D). In addition to variegated leaf sector patterning, some mature *SMD* leaves also had sporadic loss of blade expansion, which led to entire sections of *SMD* leaves failing to form laterally from the central vein (Figure 2B,C). As observed for *dem1* mutant cotyledons, the abaxial surface of mature *SMD* leaves were phenotypically indistinguishable from the abaxial surface of mature WT tomato leaves (Figure 2C,E). Furthermore, all other aspects of *SMD* vegetative development, including overall plant architecture, phyllotaxy and growth rate, were similar to these metrics documented for WT tomato plants.

Microscopy of transverse sections across the boundary of variegated sectors of mature *SMD* leaves showed that the palisade mesophyll which forms as a uniform row of columnar photosynthesis competent cells immediately below the adaxial epidermis of dark green colored WT sectors, was completely absent in the pale green colored *dem1* mutant sectors (Figure 2F). SEM of WT/*dem1* mutant sector boundaries also revealed another phenotypic similarity between *dem1* cotyledons and the *dem1* mutant sectors of mature *SMD* leaves: defective differentiation of the adaxial epidermis (Figure 2G). SEM also showed that in addition to producing adaxial epidermal cells of irregular size and shape, some of the trichomes which formed in the pale green *dem1* mutant leaf sectors developed an abnormal globular shape (Figure 2H). It is important to note here however that not all pale green *dem1* mutant leaf sectors were associated with the development of irregular adaxial epidermal cells, globular trichomes, and an absence of the palisade mesophyll (data not shown). This observation suggests that the developmental timing, and the tissue layer, where *Ds* transposition interrupted *SlDEM1* gene function influenced the degree of severity of the phenotype expressed by each *dem1* mutant leaf sector. As observed for *dem1* mutant cotyledons, SEM next revealed that abaxial leaf development, including the size and shape of epidermis cells and the stomata, was identical for dark green colored WT sectors and pale green colored *dem1* mutant sectors of mature *SMD* leaves (Figure 2I,J). In agreement with the findings stemming from analysis of *dem1* mutant seedlings (Figure 1), the phenotypic characterization of the *SMD* transformant line (Figure 2) again indicated the absolute requirement of *SlDEM1* gene function for normal cell proliferation and cell differentiation for leaf development in tomato.

**Figure 3:**
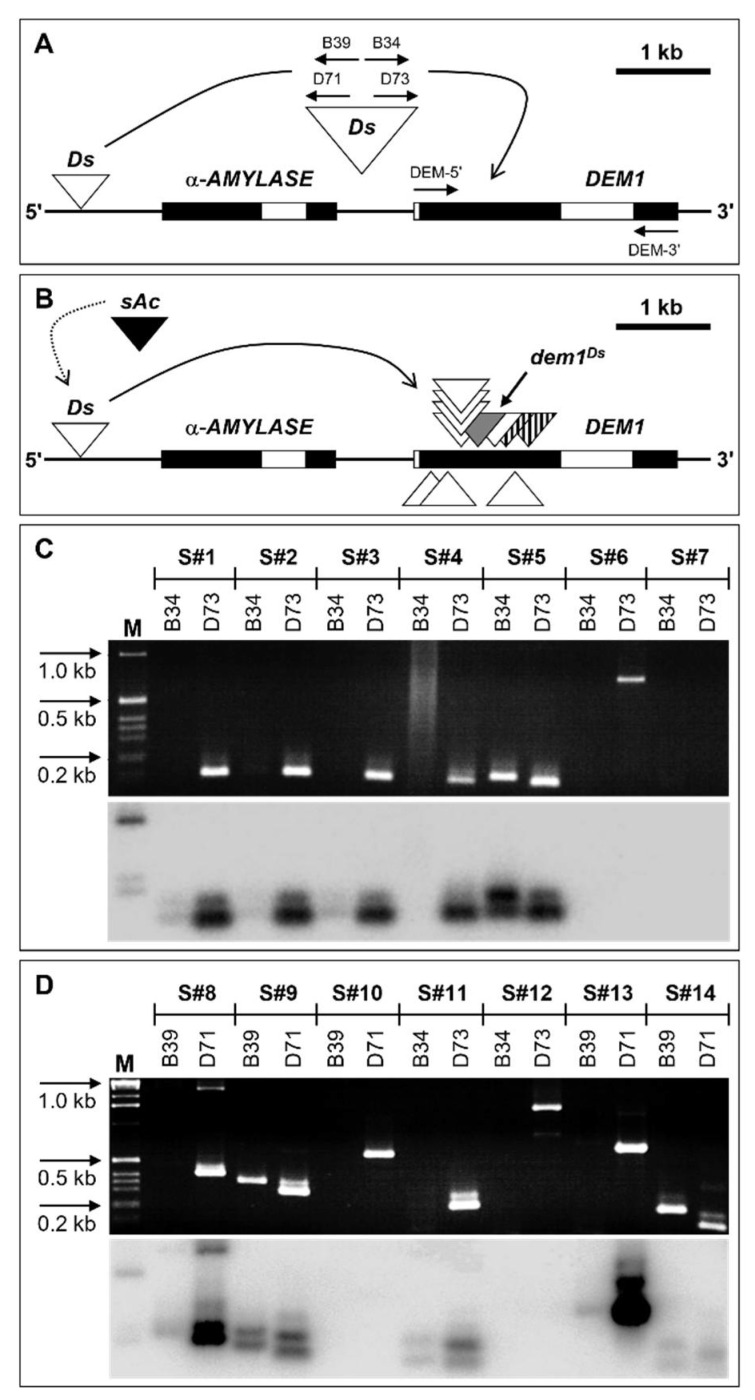
PCR-based identification of independent *Ds* insertion events into the *SlDEM1* locus in individual *dem1* mutant sectors of mature *SMD* leaves. (**A**) Schematic of the location of primers used, including the *DEM1*-specific primers, DEM5’ and DEM3′, and the *Ds*-specific primers B34, B39, D71 and D73, to detect independent *Ds* insertion events into the *SlDEM1* locus in individual *dem1* mutant sectors of *SMD* leaves. The *a-AMYLASE* gene situated between the mapped *Ds* launching pad and the *DEM1* locus in the *SMD* transformant line is also depicted with black boxes representing exons and white boxes representing introns of the α-*AMYLASE* and *DEM1* genes. (**B**) Schematic of the location (as determined by the size of individual PCR products) of individual *Ds* insertions mapped to the *SlDEM1* locus of *dem1* mutant sectors sampled from the leaves of the *SMD* transformant line with white triangles above the line representing *Ds* insertions into the forward strand of *SlDEM1* and white triangles below the line representing *Ds* insertions into the reverse strand of *SlDEM1*. The exact position of two *Ds* insertions into the *DEM1* locus was verified by sequencing of cloned PCR products as represented by triangles with vertical lines. The grey shaded triangle represents the position of a mapped *Ds* element into the *DEM1* gene (termed the *dem^Ds^* insertion) [41] which was included in this analysis as a positive control for *Ds* insertion into *SlDEM1*. (**C**) For samples S#1 to S#7, primer DEM5’ was used together with primers B34 (primary PCR) and D73 (semi-nested PCR) for amplicon production. (**D**) The DEM5’ primer was also used for PCR product amplification from samples S#8 to S#10 together with primers B39 (primary PCR) and D71 (semi-nested PCR). The primer, DEM3′, together with primers B34 (primary PCR) and D73 (semi-nested PCR), were used for amplicon generation from samples, S#11 and S#12, and for samples S#13 and S#14, the DEM3′ primer was used together with the B39 (primary PCR) and D71 (semi-nested PCR) primers to generate PCR amplicons. In (**C**,**D**) the upper panel is a photograph of an ethidium bromide-stained agarose gel under UV-light, while the lower panel represents a Southern blotted membrane post hybridization with a *SlDEM1*-specific probe.

Considering that the pale green colored sectors on the adaxial surface of *SMD* leaves was the most frequently observed phenotypic consequence of the somatic mutagenesis of the *SlDEM1* locus (Figure 2B), we next employed a semi-nested PCR approach together with the Southern blot hybridization technique to demonstrate that in each *dem1* mutant sector of a *SMD* leaf, the *SlDEM1* coding sequence harbored a mutation resulting from *Ds* transposition. To achieve this goal, a series of DNA oligonucleotide primers specific to either the *SlDEM1* locus (DEM5′ and DEM3′) or the *Ds* transposon (B34, B39, D71 and D73) were developed to facilitate the identification of unique *Ds* transposition events into the tomato *DEM1* gene in each analyzed *dem1* mutant sector of mature *SMD* leaves (Figure 3A). Furthermore, the size of the resulting amplicons produced when using each primer combination allowed for the ‘rough’ determination of the position of the *Ds* element within the *SlDEM1* gene as well as to orientate each *Ds* insertion (Figure 3B). In total, 14 genomic DNA extractions were screened via this PCR-based approach, and included; (1) a single sector of *SMD* leaf of WT appearance (sample S#7), which was included in this analysis as a negative control for *Ds* transposition into the *SlDEM1* locus; (2) two *dem1* mutant sectors (samples S#5 and S#14) sampled as positive controls from the *SMD* heterozygous plant line, *dem^Ds^*, known to harbor a stabilized *Ds* insertion in the *SlDEM1* gene (Figure 3B), and; (3) 11 pale green colored *dem1* mutant sectors (samples S#1–S#4, S#6, and S#8–S#13) sampled from *SMD* leaves to attempt to identify *SlDEM1*/*Ds*-specific PCR products. More specifically, samples S#1 to S#7 were analyzed using primer DEM5′ together with the B34 (primary PCR) and D73 (semi-nested PCR) primers (Figure 3C), while samples S#8 to S#10 were also assessed with primer DEM5′, but in combination with the B39 (primary PCR) and D71 (semi-nested PCR) primers (Figure 3D). Alternatively, samples S#11 and S#12 were screened with the *SlDEM1*-specific primer, DEM3′, and the *Ds*-specific primers, B34 (primary PCR) and D73 (semi-nested PCR), and samples S#13 and S#14 were assessed with primers DEM3′, B39 and D71, to generate PCR amplicons for analysis (Figure 3D).

The schematic presented in Figure 3B depicts the approximate location of each *Ds* insertion event that was successfully mapped to the *SlDEM1* locus with 7 and 3 of the 10 mapped *Ds* insertions determined to have inserted into the sense and antisense strand of the *SlDEM1* gene, respectively. In addition, and as expected, we failed to amplify a *DEM1*/*Ds*-specific PCR product from the *SMD* leaf sector of WT appearance, sample S#7, included in this analysis as a negative control for *Ds* transposition into the *SlDEM1* gene (Figure 3C). However, amplicons were readily amplified by PCR from the two *dem^Ds^* leaf sector samples, specifically the S#5 and S#14 samples, included in the PCR screening of *SMD* leaf sectors as positive controls for *Ds* insertion into *SlDEM1* (Figure 3C,D). The insertion of the *Ds* element into *SlDEM1* was further confirmed via sequencing of two of the products amplified by PCR from the genomic DNA extracted from two different *dem1* mutant sectors of a *SMD* leaf (Figure 3B). We next used the Southern blot hybridization technique to further assess the authenticity of each amplified PCR product (Figure 3C,D). Via the use of a probe specific to the full length complementary DNA (cDNA) sequence of the messenger RNA (mRNA) transcribed from the *SlDEM1* gene, hybridization products were obtained for samples, S#1, S#2, S#3, S#4, S#5 (positive control), S#8, S#9, S#11, S#13 and S#14 (positive control) (Figure 3C,D). Southern blot hybridization products failed to be detected for sample S#7, the negative control sample, and for *dem1* mutant sector samples, S#6, S#10 and S#12 (Figure 3C,D). The success of generating semi-nested PCR amplicons for samples S#6, S#10, and S#12, combined with our failure to obtain a *slDEM1*-specific Southern blot hybridization product for these three *dem1* mutant sector samples, may represent PCR artefacts. Nevertheless, each of these three analyzed mutant sectors most likely resulted from a *Ds* insertion into *slDEM1*, followed by the *Ds* subsequently excising out of the *SlDEM1* gene to create *dem1* frameshift mutant sectors.

### 2.3. The in Planta Expression of SlDEM1-Specific Sequences Negatively Impacted Cell Proliferation in Tomato Leaves and Failed to Complement the Developmental Phenotype Displayed by dem1 Mutant Seedlings

**Figure 4:**
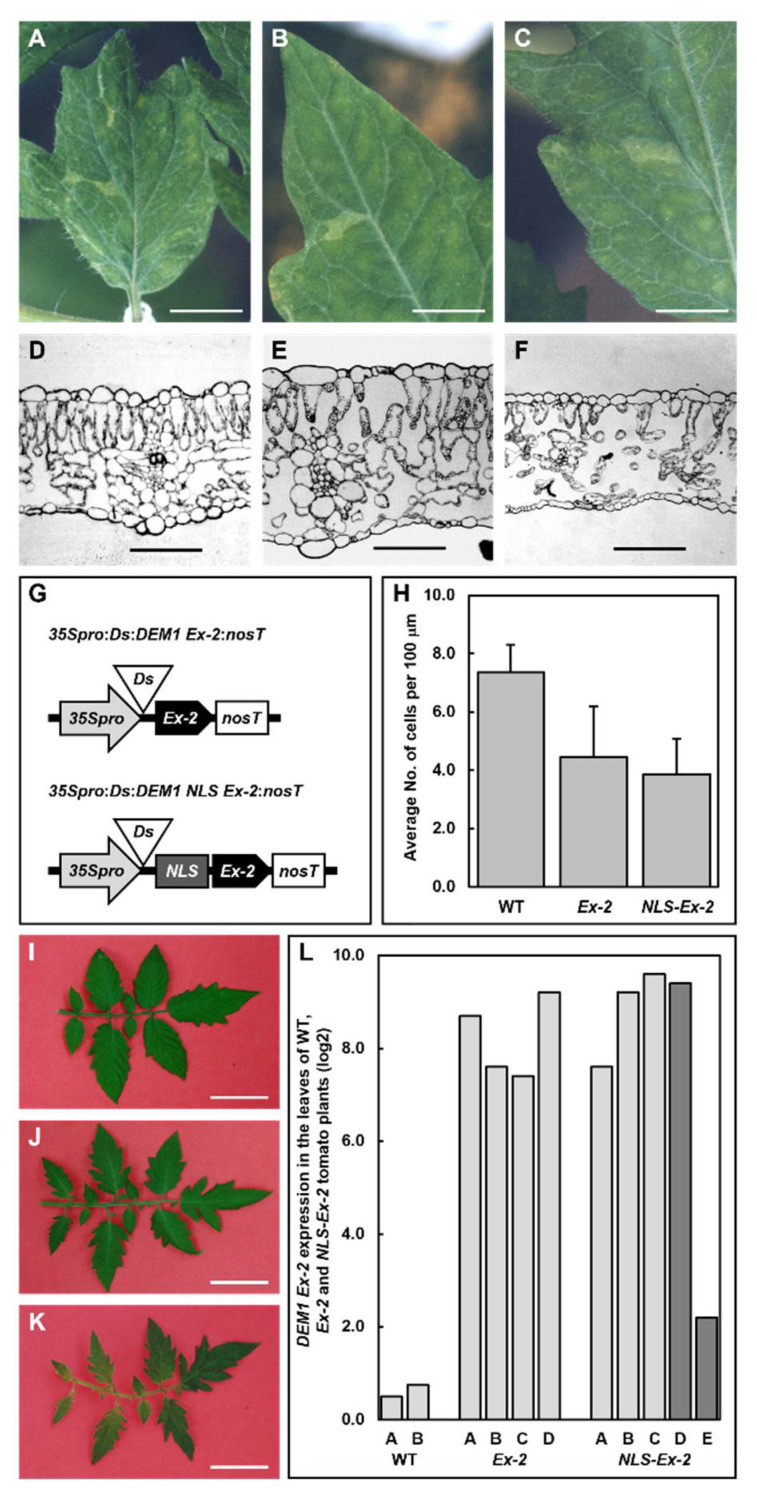
Phenotypes displayed by the *Ex-2* and *NLS-Ex-2* tomato transformant lines. (**A**–**C**) Tomato transformant lines constructed to somatically overexpress *DEM1* exon-2 only (*Ex-2* plants) or *DEM1* exon-2 together with the putative nucleus localization signal (NLS) of *DEM1* (*NLS-Ex-2* plants) developed leaf sectors similar to those observed for *SMD* leaves. (**D**–**F**) Transverse sections of *dem1* mutant sectors of *Ex-2* (**E**) and *NLS-Ex-2* (**F**) leaves clearly show that palisade mesophyll cell formation is intermittent, and that spongy mesophyll proliferation is almost absent, compared to the uniform formation of these two cell types in WT tomato leaves (**D**). (**G**) Schematic of the *DEM1* overexpression transgenes *35Spro*:*Ds*:*DEM1 Ex-2*:*nosT* and *35Spro*:*Ds*:*NLS DEM1 Ex-2*:*nosT* introduced into tomato plants to generate the *Ex-2* and *NLS-Ex-2* transformant lines, respectively. (**H**) Adaxial epidermal cell counts per 100 μm section across a 600 μm interval revealed that cell size was reduced (not statistically significant) in *dem1* mutant sectors of *Ex-2* and *NLS-Ex-2* leaves compared to WT leaf sectors. (**I**) The majority of *Ex-2* and *NLS-Ex-2* transformants which stably overexpressed *DEM1*-derived sequences developed leaves of WT appearance. (**J**) A small number of stable *Ex-2* transformants did however develop leaves with a decreased blade width and deeper degrees of margin serration. (**K**) Similarly, a small number of stable *NLS-Ex-2* transformants developed abnormal leaves with wrinkled blades, dull green coloration, and deeply serrated margins. (**L**) RT-qPCR revealed that the degree of *SlDEM1* overexpression in *Ex-2* and *NLS-Ex-2* transformant lines did not correlate with the level of severity of the leaf phenotype displayed by some *NLS-Ex-2* transformants (dark grey columns) when compared to the level of *DEM1* overexpression in stable *Ex-2* and *NLS-Ex-2* transformants which did not display leaf phenotypes (light grey columns). Scale bars = 1000 μm (**A**), 500 μm (**B**), 250 μm (**C**), 100 μm (**D**–**F**), and 2.5 cm (**I**,**J**).

Comparison of the amino acid sequence of *SlDEM1* to the DEM-like proteins of other plant species [41,42] revealed a high level of conservation of exon 2 (*Ex-2*), a finding which led us to hypothesize that this exon may potentially direct the primary function of *SlDEM1* in cell proliferation and cell differentiation in leaf development. To address this hypothesis, we constructed plant expression vector, *Ex-2*, where the *DEM1 Ex-2* sequence was cloned from tomato and placed behind the *Cauliflower mosaic virus* (CaMV) *35S* promoter (*35Spro*) to drive the *in planta* expression of the introduced sequence. Furthermore, the *Ds* element was placed between the *35Spro* and *Ex-2* sequences to interrupt transgene expression and enable study of the role of the overexpressed *SlDEM1*-derived sequence during leaf development in a genetic background which was not negatively impacted by developmental abnormalities stemming from *SlDEM1* misexpression during embryonic development (Figure 4A–G). In addition to the *Ex-2* plant expression vector, a second *DEM1*-derived plant expression vector, termed *NLS-Ex-2*, was developed to further assess the role of *SlDEM1* in leaf development in tomato. To construct this vector, the 3′ half of the first exon of the tomato *DEM1* gene was fused to the *Ex-2* sequence, an approach which was adopted due to our protein sequence analyses further indicating that this region of the *SlDEM1* coding sequence could encode for a putative nucleus localization signal (NLS) [42]. As for the *Ex-2* vector, the *in planta* expression of the *SlDEM1* derived sequences by the *35Spro* in the *NLS-Ex-2* plant expression vector was interrupted by the insertion of the *Ds* element between the *35Spro* and *NLS-Ex-2* sequences (Figure 4A–F). Following the introduction of the *Ex-2* and *NLS-Ex-2* transgenes into WT tomato plants (Figure 4G), the resulting transformant lines were genetically crossed with the *sAc* transformant line to generate offspring where the *Ex-2* and *NLS-Ex-2* transgenes were only expressed post *Ds* transposition in leaf sectors.

As observed for the *SMD* transformant line, the somatic overexpression of the *SlDEM1*-derived *Ex-2* and *NLS-Ex-2* sequences in tomato resulted in the development of pale green colored sectors on the adaxial surface of the leaves of some *Ex-2* and *NLS-Ex-2* transformants (Figure 4A–C). Comparison of the transverse sections sampled from the dark green colored sectors of *Ex-2* and *NLS-Ex-2* leaves of WT appearance (Figure 4D), to the pale green colored *dem1* mutant sectors of the same leaves (Figure 4E,F), revealed that immediately beneath the adaxial epidermis of *dem1* mutant sectors, the formation of the palisade mesophyll was either highly disorganized or almost failed to form. Furthermore, the formation of the spongy mesophyll was greatly reduced in the abaxial half of *dem1* mutant sectors of the leaves of *Ex-2* and *NLS-Ex-2* transformants with very large airspaces forming the majority of the lower half of the leaf in *dem1* mutant sectors (Figure 4E,F). This phenotype is in direct contrast to those of either the abaxial half of *dem1* cotyledons (Figure 1G), or *dem1* mutant sectors of *SMD* leaves (Figure 2F), where spongy mesophyll cell proliferation appeared to be promoted, leading to a reduction to both the size and frequency of the airspaces which formed above the abaxial epidermis of WT tomato cotyledons (Figure 1F), or the dark green colored WT sectors of *SMD* leaves (Figure 2F). The somatic overexpression of the *SlDEM1*-derived *Ex-2* and *NLS-Ex-2* sequences did not negatively impact abaxial epidermis development (data not shown) as observed for *dem1* mutant cotyledons and *dem1* mutant sectors *SMD* leaves (Figure 2I,J). However, abnormal adaxial epidermis development was further confirmed for the *Ex-2* and *NLS-Ex-2* transformant lines via performing cell counts per 100 micrometer (μm) sections across 600 μm intervals of WT sectors, and *dem1* mutant sectors of *Ex-2* and *NLS-Ex-2* leaves (Figure 4H). This analysis revealed that compared to the WT sectors of *Ex-2* and *NLS-Ex-2* leaves, adaxial epidermal cell size was reduced, albeit not significantly, in the *dem1* mutant sectors of the same leaves sampled from *Ex-2* and *NLS-Ex-2* plants (Figure 4H).

Stable transposition of the *Ds* element from its original launching pad position between the *35Spro* and the *SlDEM1*-derived *Ex-2* and *NLS-Ex-2* sequences was confirmed for some *Ex-2* and *NLS-Ex-2* transformant lines. The majority of these transformants developed leaves of WT appearance (Figure 4I), however, a small number of stable *Ex-2* transformants did develop leaves with decreased blade width and higher degrees of margin serration, and which displayed a slightly paler green coloration (Figure 4J). Similarly, only a small subset of stable *NLS-Ex-2* transformant lines displayed a leaf development phenotype which was characterized by an overall reduction in leaf size due to a reduction in both the length and width of blades; leaf blades which also developed highly serrated margins and mottling from intermittent sectors of pale and dark green coloration (Figure 4K). Leaves were next sampled from stable *Ex-2* and *NLS-Ex-2* transformants that did (Figure 4J,K), and did not display leaf phenotypes (Figure 4I), with the sampled tissue subsequently used for total RNA extraction and cDNA synthesis. Interestingly, reverse transcriptase quantitative polymerase chain reaction (RT-qPCR) analysis revealed no correlation between the level of *SlDEM1* sequence overexpression and the degree of severity of the leaf development phenotypes displayed by stable *Ex-2* and *NLS-Ex-2* transformant lines (Figure 4L). More specifically, RT-qPCR showed that the abundance of the *SlDEM1* transcript was much greater in the leaves of transformant line *NLS-Ex-2* C in Figure 4L, which expressed a WT-like phenotype (Figure 4I), than the level of expression of the *SlDEM1* transcript in the leaves sampled from the *NLS-Ex-2* E transformant line (Figure 4L), which displayed a severe leaf development phenotype (Figure 4K).

**Figure 5:**
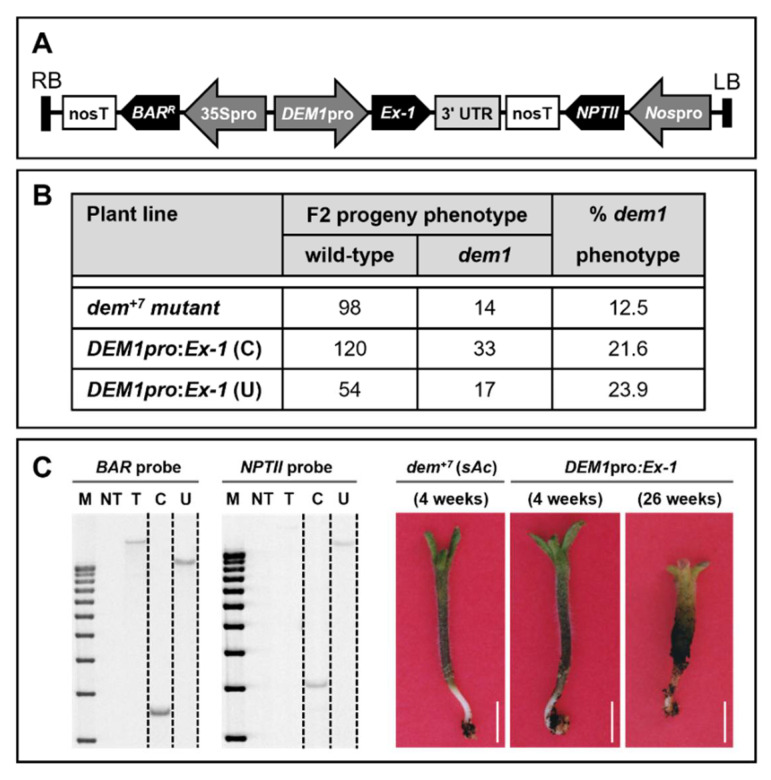
The *in planta* expression of the *DEM1pro:Ex-1* transgene failed to complement the *dem1* mutant phenotype. (**A**) The *dem^+7^* mutant was genetically crossed with the *DEM1pro:Ex-1* transformant line which harbored the *DEM1pro:Ex-1* transgene and the resulting progeny plants were allowed to self-fertilize. Key: RB, T-DNA right border; *nosT*, *nopaline synthase* terminator; *BAR^R^*, *bialaphos* resistance gene; *35Spro*, CaMV 35S promoter; *DEM1pro*, *DEM1* promoter; *Ex-1*, *DEM1* exon 1; 3′ UTR, *DEM1* 3′ untranslated region; *NPTII*, neomycin phosphotransferase; *Nospro*, *nopaline synthase* promoter; LB, T-DNA left border. (**B**) The rate of germination of seedlings which expressed the *dem^+7^* mutant phenotype was increased to near the expected Mendelian ratio of 3:1 (wild-type to mutant) in the F2 progeny of *DEM1pro:Ex-1* transgene single copy transformant lines post their genetic crossing with the *dem^+7^* mutant. (**C**) Southern blot hybridization analysis of *DEM1pro:Ex-1* transformant lines using probes specific to the *BAR^R^* and *NPTII* genes harbored by the *DEM1pro*:*Ex-1* transgene to demonstrate that both transformant lines only harbor a single copy of the introduced transgene. Key: M, DNA ladder; NT, no template control; T, template control; C, single copy *DEM1pro*:*Ex-1* transformant line (C); U, single copy *DEM1pro*:*Ex-1* transformant line (U); dashed lines depict cropping and merging of hybridized filters to only show these two single copy lines as examples. No phenotypic difference was observed between the mutant progeny of *dem^+7^* plants, and the *dem^+7^* mutant phenotype expressing *DEM1pro*:*Ex-1* (C) and *DEM1pro*:*Ex-1* (U) transformant lines at 4 weeks of age. At 6 months of age (26 weeks), the *DEM1pro*:*Ex-1* (C) and *DEM1pro*:*Ex-1* (U) transformant lines that expressed the *dem^+7^* mutant phenotype still failed to progress past the initial stages of seedling development with hypocotyl thickening and tissue yellowing the only phenotypic differences observed. Scale bar = 1.0 cm.

The more severe vegetative phenotype displayed by a small cohort of stable *NLS-Ex-2* transformants (Figure 4K), compared to the milder vegetative phenotype expressed by an equally small number of stable *Ex-2* transformants (Figure 4J), led us to next hypothesize that the *DEM1 exon-1* (*Ex-1*) sequence which harbors the putative NLS overexpressed in *NLS-Ex-2* transformants may be more crucial than the more highly conserved *Ex-2* sequence with respect to the role played by *SlDEM1* in tomato vegetative development. To test this hypothesis, we developed the *DEM1pro:Ex-1* transgene for the *in planta* expression of the *DEM1 Ex-1* sequence under the control of its own endogenous promoter (*DEM1pro*) (Figure 5A). The 3′ untranslated region (UTR) of the tomato *DEM1* gene was also included in the *DEM1pro:Ex-1* transgene in case this non-coding sequence of the *SlDEM1* locus contained any regulatory sequences essential to *DEM1* gene function in tomato vegetative development (Figure 5A). The progeny of WT tomato plants transformed with the *DEM1pro:Ex-1* transgene, and determined to be homozygous for a single copy of the inserted transgene via Southern blot hybridization analysis using probes specific to the *NPTII* and *BASTA* selectable marker genes included in the *DEM1pro:Ex-1* transgene (Figure 5C), were genetically crossed with a second tomato line which harbored a stable, heterozygous *dem^+7^* mutation (Figure 5A). The progeny of this cross (F1 plants) determined to be heterozygous for both the *dem^+7^* mutation and the *DEM1pro:Ex-1* transgene were allowed to self-pollinate.

When a *dem^+7^* heterozygous representative plant was self-fertilized, only 14 of the 112 (12.5%) seedlings which germinated expressed the *dem1* mutant phenotype. A phenotype expression frequency of approximately 25% (that is; a 3:1 ratio of phenotypically normal to mutant progeny plants) is expected for a single mutated allele in a diploid organism according to the principles of Mendelian inheritance. Therefore, the observed 12.5% frequency of expression of the *dem1* mutant phenotype in the seedlings of a self-fertilized *DEM1/dem^+7^* parent plant strongly suggested that mutation of the *SlDEM1* locus directed a high degree of lethality during tomato embryonic development. The introduction of the *DEM1pro:Ex-1* transgene, and its subsequent expression in the *dem^+7^* heterozygous mutant background was revealed to increase the percentage of germinated seedlings which expressed the *dem1* mutant phenotype to closer to the expected frequency of 25% in the single copy transformant lines, *DEM1pro:Ex-1* (C) and *DEM1pro:Ex-1* (U) (Figure 5B). This finding indicated that the expression of *SlDEM1 Ex-1* in the seed embryo provided a degree of promotion to embryonic tissue development resulting in the observed increase in germinated seeds which expressed the *dem1* mutant phenotype (Figure 5B,C). However, *DEM1pro:Ex-1* transgene expression failed to provide any level of complementation of the severe developmental phenotype expressed by *dem^+7^* mutant seedlings (Figure 5C). More specifically, 4-week-old *dem1* mutant seedlings of the single copy *DEM1pro:Ex-1* (C) and *DEM1pro:Ex-1* (U) transformant lines, expressed a phenotype that exactly phenocopied the phenotype displayed by 4-week-old *dem^+7^* mutant seedlings (Figure 5C). Furthermore, the only developmental progression observed in either 26-week-old *DEM1pro:Ex-1* (C) or *DEM1pro:Ex-1* (U) seedlings that expressed the *dem1* phenotype was thickening of hypocotyl girth and the further discoloration of the cells of the upper terminal region of the hypocotyl (Figure 5C). Failure of the development of *dem1* mutant phenotype expressing seedlings of either the *DEM1pro:Ex-1* (C) and *DEM1pro:Ex-1* (U) transformant line to further progress from their 4-week-old stage of development, 6 months (26 weeks) post initial seed germination, revealed that expression *SlDEM1 Ex-1* sequence failed to even partially complement the severe developmental abnormalities which result from disruption of *SlDEM1* gene function during the initial stages of early tomato development.

### 2.4. The Adaxial Tissue and Blade Expansion Defects of the Leaves of SMD Transformants Are Similar to Those Expressed by Plants with Altered PHAN and KNOX1 Gene Expression

The failure of leaf sectors to expand laterally from the central vein and the abnormal development of the adaxial tissue of the leaves of the *SMD* transformant line share a degree of similarity to the phenotypes displayed by the *Antirrhinum phantastica* (*phan*) mutant [46]: a mutant plant line characterized by meristem maintenance defects and abnormal adaxial leaf tissue development. More specifically, abaxial cells replace adaxial cells in early leaves, and later leaves which are largely composed of abaxial cell types adopt an overall needle-like shape due to their almost complete failure to expand laterally from the central vein [32,37,46]. In leaf development, the primary function of PHAN-like transcription factors is to repress the transcriptional activity of specific *KNOX1* genes to either promote or repress cell proliferation and/or cell differentiation in the SAM peripheral zone [26,28,33,34,36,39,40]. It has also been demonstrated previously that the overexpression of the KN-like transcription factors, *AtKNAT1* and *AtKNAT2* in *Arabidopsis*, leads to a decrease in the degree of palisade mesophyll differentiation and abnormal adaxial epidermal cell morphology [23,47,48]. Therefore, the similarity of the vegetative phenotypes displayed by the tomato *SMD* transformant line, the *Antirrhinum phan* mutant, and the *Arabidopsis KNOX1* gene overexpression lines, led us to next attempt to determine whether altered *SlDEM1* expression could downregulate the expression of *KNOX1* genes in tomato, possibly in cooperation with *SlPHAN*. To address this hypothesis, northern blot hybridization analysis was initially used to assess the transcript abundance of *SlPHAN*, and of the tomato *KNOX1* genes, *SlTKN1* and *SlTKN2*, in the cotyledons and hypocotyls of 4-week-old WT tomato and *dem1* mutant seedlings.

**Figure 6:**
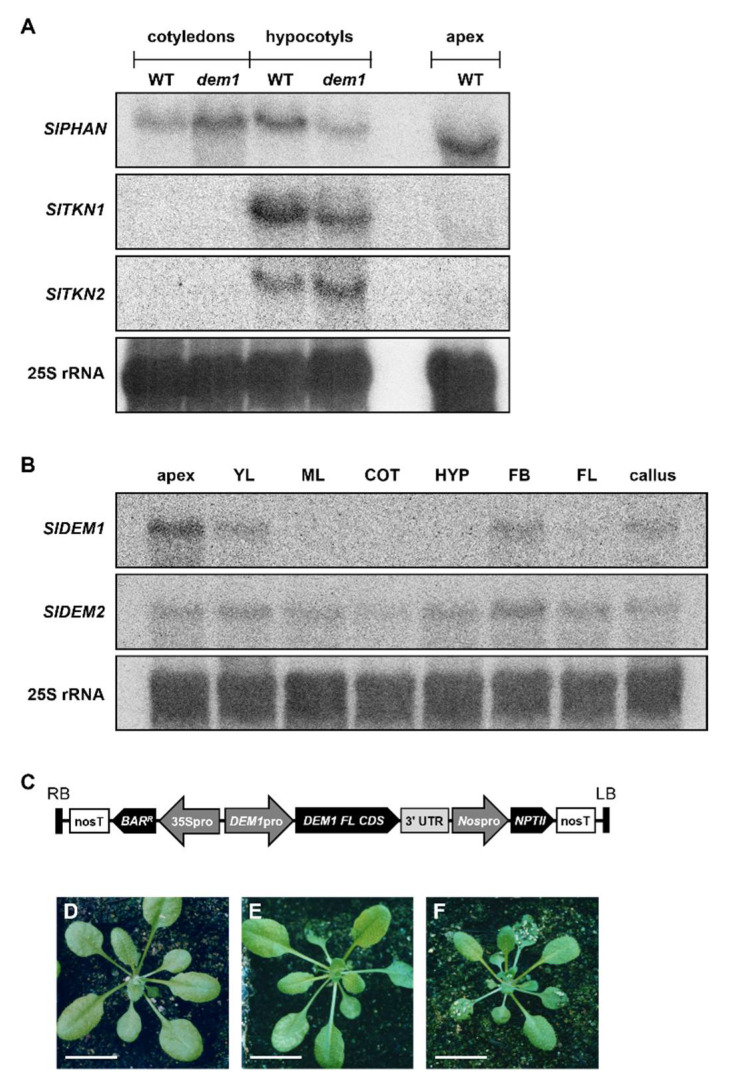
Northern blot hybridization analysis of the transcript abundance of *DEM1* and *DEM2* and of their potential interactors in WT tomato plants and the *dem1* mutant and the transformation of *Arabidopsis* with *SlDEM1* expressing transgenes. (**A**) Northern blot hybridization analysis of the expression of putative *SlDEM1* interactors, *SlPHAN*, *SlTKN1* and *SlTKN2*, in the cotyledons and hypocotyls of WT tomato plants and the *dem1* mutant. (**B**) Construction of the expression profile of *SlDEM1* and *SlDEM2* in developmentally distinct tissues of WT tomato plants via northern blot hybridization analysis. (**A**,**B**) Post the initial analysis of the expression of genes of interest, radiolabeled membranes were striped and reanalyzed with a probe specific to the 25S rRNA for use as a loading control. (**C**) Schematic of the *DEM1pro*:*DEM1-FL-CDS* transgene for the *in planta* expression of the full-length CDS and 3′ UTR of *SlDEM1* gene under the control of the *SlDEM1* promoter. Key: RB, T-DNA right border; *nosT*, *nopaline synthase* terminator; *BAR^R^*, *bialaphos* resistance gene; *35Spro*, CaMV 35S promoter; *DEM1pro*, *DEM1* promoter; *DEM1-FL-CDS*, full-length *DEM1* coding sequence; 3′ UTR: *DEM1* 3′ untranslated region; *NPTII*, *neomycin phosphotransferase* gene; *Nospro*, *nopaline synthase* promoter; LB, T-DNA left border. Wild-type *Arabidopsis* plants, ecotype Columbia-0 (Col-0) (**D**) were transformed with the *DEM1pro*:*Ex-1* (**E**) or *DEMpro*:*DEM1-FL-CDS* (**F**) transgenes. (**D**–**F**) Scale bar = 1.0 cm.

The transcript abundance of the putative *SlDEM1* interactor, *SlPHAN*, was revealed by northern blotting to be increased in the *dem1* cotyledon, but to be reduced in the *dem1* hypocotyl, compared to the abundance of *SlPHAN* in the corresponding tissues of 4-week-old WT tomato seedlings (Figure 6A). The abundance of the transcripts of the two assessed *KNOX1* genes, *SlTKN1* and *SlTKN2*, failed to accumulate to levels detectable by the standard northern blotting approach applied here in the cotyledons of 4-week-old WT tomato and *dem1* mutant seedlings. However, northern blot hybridization analysis did reveal that the abundance of the *SlTKN1* transcript was reduced in *dem1* hypocotyls compared to its expression level in WT tomato hypocotyls, whereas *SlTKN2* transcript abundance was mildly elevated in *dem1* hypocotyls compared to its abundance in the hypocotyls of 4-week-old WT tomato seedlings (Figure 6A). Failure to detect a consistent alteration to the level of expression of *SlPHAN*, *SlTKN1* and *SlTKN2* in the *dem1* mutant background indicated that there was no clear relationship between altered *SlDEM1* gene expression and the level of expression of the three assessed genes. Therefore, a yeast two-hybrid approach was next applied to determine if the *SlDEM1* protein could potentially form an interaction with either the *SlPHAN*, *SlTKN1* or *SlTKN2* proteins: an interaction which we hypothesized could occur in the apices of young tomato seedlings. However, no readily apparent protein–protein interactions were established by the yeast two-hybrid system using full length clones of the tomato DEM1, PHAN, TKN1 and TKN2 proteins (data not shown). This finding clearly indicated that in addition to an apparent lack of genetic interaction, there was also no direct protein–protein interactions between *SlDEM1* and *SlPHAN*, *SlTKN1* and *SlTKN2*.

Like most dicotyledonous plants, tomato encodes two *DEM* genes, specifically *SlDEM1* and *SlDEM2* [41,42]. Northern blot hybridization analysis was subsequently used to document the expression domains of *SlDEM1* and *SlDEM2* across a developmentally distinct set of tomato tissues: an experiment which was undertaken to attempt to determine which of the two tomato *DEM* genes potentially plays a more central role throughout the entire cycle of tomato development. Northern blotting clearly showed that the expression of *SlDEM1* is restricted to specific tissues and stages of tomato development (Figure 6B). More specifically, *SlDEM1* is highly expressed in the shoot apex (Figure 6B), and moderately expressed in young leaves and floral buds. In contrast, *SlDEM2* was revealed by northern blotting to have a more expansive expression domain in tomato with the *SlDEM2* transcript accumulating to its highest degree of abundance in floral buds, but with *SlDEM2* hybridization products also detected in the shoot apex, young and mature leaves, cotyledons, hypocotyls and flowers of WT tomato plants (Figure 6B). Interestingly, both *SlDEM1* and *SlDEM2* were determined to be expressed in the callus derived from immature embryos, a finding that further identifies an important, yet still unknown role for both *DEM* genes in the very early stages of tomato development. It is also important to note here that we have recently documented a highly similar pattern of expression for *AtDEM1* and *AtDEM2* throughout *Arabidopsis* development [42]. More specifically, *AtDEM1* was determined to have a more restricted range of expression than the *AtDEM2* gene, with the highest degree of *AtDEM1* expression detected in the reproductive tissues of *Arabidopsis*, namely the floral buds, flowers and siliques. In contrast, RT-qPCR revealed *AtDEM2* to be more highly expressed than *AtDEM1* in all assessed *Arabidopsis* tissues, including the shoot apex, young and mature leaves, the inflorescence, floral buds, flowers and siliques [42].

The shared transcriptional relationship between *SlDEM1* and *SlDEM2* in tomato, and *AtDEM1* and *AtDEM2* in *Arabidopsis*, led us to next transform WT *Arabidopsis* plants (ecotype Columbia-0 (Col-0)) with two plant expression vectors that directed the *in planta* expression of *SlDEM1*-derived sequences to determine whether such a heterologous approach could alter either the vegetative or reproductive phase of *Arabidopsis* development. The first plant expression vector introduced into *Arabidopsis* was the *DEM1pro:Ex-1* vector (Figure 5A), a vector that was initially developed to attempt to complement the severe developmental phenotype of the tomato *dem1* mutant (Figure 5C). For the second plant transformation vector, termed the *DEM1*pro:*DEM1*-*FL-CDS* vector, the *SlDEM1 Ex-1* sequence of the *DEM1pro:Ex-1* transgene which was positioned between the endogenous promoter (*DEM1pro*) and 3′ UTR of the tomato *DEM1* gene, was replaced with the full-length coding sequence (*FL-CDS*) of *SlDEM1* (*DEM1-FL-CDS*) (Figure 6C). The homozygous T2 progeny of *Arabidopsis* transformant lines determined to harbor a single copy of either the *DEM1pro:Ex-1* (Figure 6E) or *DEM1pro:DEM1-FL-CDS* (Figure 6F) transgene displayed rosette leaf morphology identical to the morphology of rosette leaves of WT Col-0 plants (Figure 6D). However, the overall size of the rosette of *DEM1pro:DEM1-FL-CDS* transformants (Figure 6F) was reduced compared to the rosettes of Col-0 plants (Figure 6D) or the *DEM1pro:Ex-1* transformant line (Figure 6E). Despite the reduced size of *DEM1pro:DEM1-FL-CDS* rosettes (Figure 6F), all other aspects of the vegetative and reproductive development of the *DEM1pro:Ex-1* and *DEM1pro:DEM1-FL-CDS* transformant lines matched those displayed by Col-0 plants. The introduction of an additional copy and expression of *SlDEM1*-derived sequences on top of the endogenous levels of *AtDEM1* and *AtDEM2* expression in *Arabidopsis* was hypothesized to alter *Arabidopsis* development, however, the WT-like phenotypes displayed by the *Arabidopsis DEM1pro*:*Ex-1* and *DEM1pro*:*DEM1-FL-CDS* transformants (Figure 6D–F) indicated that the tomato *DEM1* gene failed to direct a similar role in early *Arabidopsis* vegetative development as it does in the early stages of tomato vegetative development.

## 3. Discussion

### 3.1. The dem1 Mutant and SMD Transformant line Show That DEM1 Is Required for Cell Proliferation and Cell Differentiation during Tomato Vegetative Development

In plants, cell proliferation and cell differentiation are the sole determinants of the final form that each adult tissue adopts with the integration of these two processes forming an absolute requirement for the development of organs of the correct size, shape, and order [49,50,51]. Like all dicotyledonous plant species, wild-type tomato seedlings produce two cotyledons, whereas *dem1* seedlings frequently developed three cotyledons of greatly reduced overall size (Figure 1A). Indeed, *dem1* seedlings have also been observed to produce between one to five small sized and incorrectly shaped cotyledons [41] with SEM analysis of sections through the apices of *dem1* seedlings revealing that an organized SAM is absent and leaf primordia initiation is defective in this mutant background (Figure 1B,C) [41]. Microscopy additionally showed that apical disorganization extended into the upper half of *dem1* mutant cotyledons as evidenced by the small size and irregular shape of the adaxial epidermis cells (Figure 1D,E). Beneath the abnormal adaxial epidermis of *dem1* mutant cotyledons, the number of palisade mesophyll cells was reduced and those cells that formed were variable in size and shape compared to the uniform columnar shape of palisade mesophyll cells of WT tomato cotyledons (Figure 1F,G). SEM of the same transverse sections further showed that *dem1* mutant cotyledons were thicker than those of WT tomato seedlings due to the enhanced proliferation of the spongy mesophyll in the abaxial half of *dem1* cotyledons: an enhancement to cell proliferation which expanded the location of this cell type into the adaxial half of *dem1* cotyledons and which greatly reduced both the size and frequency of the air spaces which uniformly form in the abaxial half of WT tomato cotyledons (Figure 1F,G). Although hypocotyl development was revealed to be normal in the *dem1* mutant, an observation which strongly suggests that disruption of *SlDEM1* gene function only affects specific aspects of embryonic development, apical growth was revealed to terminate soon after germination with no true leaf or root structures forming in *dem1* seedlings due to an inability of the mutant to initiate and maintain shoot or root meristems during embryonic development [41]. Tomato mutant plant lines with SAM initiation or maintenance defects [52], which fail to establish leaf primordia [53], that produce an incorrect number of cotyledons [54], or which have defective adaxial epidermis [40] or mesophyll cell development [55], have all been reported previously. However, to the best of our knowledge, the *dem1* mutant is the first reported mutant in tomato where all of these essential developmental processes are defective.

The seedling lethality of the *dem1* mutant led to the development of the *SMD* transformant line to assess the involvement of the *SlDEM1* gene in the subsequent stages of tomato vegetative development. Figure 2B,C show one of the two most striking phenotypes displayed by *SMD* transformants resulting from *Ds*-directed disruption of *SlDEM1* gene function post the seedling stage of vegetative development in tomato, specifically, termination of cell proliferation leading to the sporadic loss of leaf blade expansion from the midvein of *SMD* leaves, an aspect of leaf development long proposed to require the juxtaposition of adaxial and abaxial cell types [32]. Furthermore, this phenotype displayed by *SMD* leaves confirmed the observations made in *dem1* mutant seedlings (Figure 1) that *SlDEM1* gene function is essential for cell proliferation during the vegetative phase of tomato development (Figure 2). The second readily apparent phenotypic consequence of *Ds*-directed disruption to *SlDEM1* gene function during tomato vegetative development was the light green colored sectors which formed on *SMD* leaves, leaf sectors which appeared to be thinner than the dark green colored sectors of WT appearance which formed on the same leaves of *SMD* transformants (Figure 2B–E). Transverse sections across WT/*dem1* mutant sectors of *SMD* leaves revealed that the ‘thinness’ of *dem1* mutant sectors was the result of defective palisade mesophyll cell proliferation (Figure 2F). Figure 2F also shows, and as observed in *dem1* mutant cotyledons (Figure 1G), that spongy mesophyll development was promoted in *dem1* mutant sectors of *SMD* leaves, with the promoted cell type extending from the abaxial to the adaxial half of *dem1* mutant leaf sectors. The formation of globular shaped trichomes and small and irregularly shaped cells on the adaxial epidermis (Figure 2H), together with the WT development of the abaxial epidermis of *dem1* mutant sectors of *SMD* leaves (Figure 2I,J), further confirmed that the function of the *SlDEM1* gene is restricted to specific aspects of embryogenic and vegetative development in tomato.

### 3.2. Molecular Manipulation of DEM1 Gene Expression Confirms the Requirement of SlDEM1 for Cell Proliferation and Cell Differentiation in Tomato

Considering that the *DEM1* gene of tomato, and the *DEM-LIKE* genes of other plant species encode for proteins of no known biochemical function, the coding sequence of the most highly conserved region of the *SlDEM1* protein, exon-2 (*Ex-2*) [41,42], was selected for somatic overexpression in WT tomato plants to further confirm the requirement of *SlDEM1* for normal embryogenic and adaxial leaf tissue development. Protein sequence analysis of *DEM1-LIKE* genes across a range of higher plant species revealed that in addition to *Ex-2*, the second half of exon-1 (*Ex-1*) encodes a highly conserved 35 amino acid motif [41,42]. Although this highly conserved motif was found not to have homology to any characterized functional domains of plant proteins, the region was determined to be highly homologous to a fission yeast (*Schizosaccharomyces pombe*) amino acid sequence which directs protein expression to the nuclear rim of yeast cells [56]. This sequence was therefore fused to the *SlDEM1 Ex-2* sequence to form the *NLS-Ex-2* transgene to determine whether the encoded motif acted as a nucleus localization signal (NLS) for the *SlDEM1* protein in tomato. The somatic overexpression of *SlDEM1 Ex-2* with (*NLS-Ex-2* plants) and without (*Ex-2* plants) the putative NLS again resulted in the development of *dem1* mutant sectors in some leaves of *Ex-2* and *NLS-Ex-2* transformants (Figure 4), as was observed for the *SMD* transformant line where the expression of *SlDEM1* had been somatically disrupted (Figure 2). As observed in the *SMD* transformant line (Figure 2F), transverse sections of *dem1* mutant sectors of *Ex-2* and *NLS-Ex-2* leaves revealed that such sectors were primarily characterized by a high degree of repression to the proliferation of palisade mesophyll cells (Figure 4E,F). The degree of repression to palisade mesophyll cell proliferation tended to be more severe in the *dem1* mutant sectors of *NLS-Ex-2* leaves than in *Ex-2* leaves (Figure 4E,F). However, it is important to note here that the palisade cells which did form in the *dem1* mutant sectors of transformant lines expressing either transgene adopted a similar shape to WT palisade mesophyll cells (Figure 4D–F). This finding indicated that the somatic overexpression of *SlDEM1*-derived sequences had a stronger influence on cell proliferation than on cell differentiation in the adaxial half of tomato leaves. In addition, and in direct contrast to the promotion of proliferation of spongy mesophyll cells readily observed in *dem1* mutant cotyledons (Figure 1G), and to a lesser degree in the adaxial half of *dem1* mutant sectors of *SMD* leaves (Figure 2F), the spongy mesophyll almost completely failed to form in the abaxial regions of the *dem1* mutant sectors of *Ex-2* and *NLS-Ex-2* leaves (Figure 4E,F). Taken together, these results firmly identified a repressive role for the highly conserved *Ex-2* region of the *DEM1* gene in mesophyll cell proliferation in tomato leaves.

The protein sequence analyses [42] further revealed that unlike the high level of conservation of *Ex-2* of *DEM1-LIKE* genes or the putative NLS motif encoded by the 3′ end of *Ex-1* of *DEM1-LIKE* genes across a range of plant species, the amino terminal region of *Ex-1* of *DEM1-LIKE* genes is much more variable in its sequence composition. Due to the high degree of sequence variability of the amino terminal region of *Ex-1* of the assessed *DEM1-LIKE* genes [42], it was hypothesized that this region may potentially encode for a function specific to the DEM1 protein of each assessed species. The introduction of an additional copy of the *SlDEM1 Ex-1* sequence to the tomato genome whose expression was under the control of the endogenous *SlDEM1* promoter failed to alter the phenotypic properties of tomato during either the vegetative or reproductive phase of development. However, genetic crossing of the generated *Ex-1* transformant line to the *dem^+7^* mutant line was revealed to promote embryonic development via returning the frequency of expression of the *dem1* mutant phenotype in the progeny resulting from this genetic cross closer to the expected frequency of 25% for a mutant phenotype resulting from the disruption of a single gene in a diploid organism (Figure 5B). Although the *in planta* expression of an additional copy of the amino terminal region of *SlDEM1 Ex-1* improved the poor embryonic performance of the *dem^+7^* mutant, as evidenced by the almost doubling of the rate of germination of *dem1* mutant phenotype expressing seedlings (Figure 5B), the failure of these *dem1* mutant seedlings to progress to a subsequent stage of vegetative development (Figure 5C), clearly revealed that either additional *SlDEM1* gene coding sequences, and/or the inclusion of specific regulatory regions surrounding the tomato *DEM1* locus, are required to provide any level of meristem function, correct cotyledon differentiation, or for leaf primordia initiation to achieve full complementation of the phenotypic consequence of *SlDEM1* gene dysfunction during the very early stages of tomato vegetative development.

The somatic mutagenesis of the *SlDEM1* gene (Figure 2), or the sectored (Figure 4) or stable (Figure 5) expression of specific regions of the *SlDEM1* gene as transgenes clearly demonstrates the value of the use of such a combinatorial approach to further study the function of developmentally important genes whose dysfunction results in the expression of severe to lethal developmental phenotypes (Figure 1). Via such an approach, here we have been able to assign new roles to the tomato *DEM1* gene in cell proliferation and cell differentiation. Some genes have been assigned clear roles in both processes, such as the *SUPERMAN* and *SCHIZOID* genes of *Arabidopsis* [57,58], whereas the functional role of other developmental genes has been shown to be specific to only one of these two processes. For example, the maize *tangled1* and *warty-1* mutants both show cell division defects while the affected cells, and therefore tissues and organs, differentiate relatively normally [50,59,60]. However, and relating directly to the severity of the expressed developmental phenotype, assignment of a specific function to a gene in one or both of these processes can prove challenging to interpret when differentiation is altered sufficiently to cause major changes to the arrangement of tissues in an organ, such as the challenges posed with assigning primary function to a gene in mutant plant lines such as the *phan* mutant of *Antirrhinum* [32,46] or the *phabulosa* and *pinhead*/*zwille* mutants of *Arabidopsis* [61,62]. In all three mutant plant lines, the development of the different cell types which comprise the tissue along the dorsoventral axis is affected by loss of function of each of these genes leading to gross changes to overall leaf size and shape, as well as to also drastically alter the arrangement of each tissue of the leaves or leaf-like structures which form in the *phan*, *phabulosa* and *pinhead*/*zwille* mutant backgrounds [32,46,61,62]. In summary, via our unique combination of the use of somatic mutagenesis and transgene-based approaches, we have been able to assign new developmental functions to the tomato *DEM1* gene, including (1) lateral expansion of the leaf blade (Figure 2B,C), (2) adaxial epidermal cell differentiation (Figure 2H), (3) adaxial trichome development (Figure 2H), (4) palisade mesophyll cell proliferation (Figure 2F), and (5) spongy mesophyll cell proliferation (Figure 4E,F). In addition to playing these specific roles in tomato leaf development, molecular modification of *SlDEM1* gene expression identified a further role for the tomato *DEM1* gene in promoting embryo health (Figure 5B), most likely by improving the fitness of the embryo during the very early stages of embryo development prior to the formation of leaf primordia.

### 3.3. In Tomato, DEM1 Does Not Directly Interact with KNOX1 or PHAN-LIKE Genes at Either the Genetic or Molecular Level

The similarities of the phenotypes expressed by the *dem1* mutant and the *SMD*, *Ex-2* and *NLS-Ex-2* transformant lines in tomato, to those expressed by the *Antirrhinum phan* mutant [32,37,46] and the *Arabidopsis* transformant lines molecularly manipulated to overexpress the *Arabidopsis KNOX1* genes, *KNAT1* and *KNAT2* [23,47,48], prompted us to attempt to uncover any alteration to the transcriptional activity of the tomato orthologs of *AmPHAN*, *AtKNAT1* and *AtKNAT2* which stemmed from the loss of *SlDEM1* gene function. However, northern blot hybridization analysis clearly revealed a lack of genetic relationship between the *SlPHAN*, *SlTKN1* and *SlTKN2* transcripts in the cotyledons and hypocotyls of WT tomato and *dem1* mutant seedlings (Figure 6A), with the lack of interaction between *SlDEM1* and *SlPHAN*, *SlTKN1* and *SlTKN2* also confirmed at the protein level via the use of the yeast two-hybrid system (data not shown). The appropriate regulation of *KNOX1* gene expression is essential for meristem maintenance, leaf initiation, and the control of the development of compound leaves in species such as tomato [40,63] with research conducted on simple leaf species such as maize, *Antirrhinum* and *Arabidopsis* revealing *KNOX1* gene expression patterns to be complementary to those of *PHAN-LIKE* genes, more specifically; *KNOX1* genes are expressed throughout the SAM except in initiating leaf primordia where *PHAN-LIKE* genes are highly expressed [32,33,34,39]. Such molecular interplay between *PHAN-LIKE* and *KNOX1* genes has been most thoroughly characterized in *Arabidopsis* where the transcriptional activity of the *AmPHAN* ortholog, *ASYMMETRIC LEAVES1* (*AS1*), is negatively regulated by the *ZmKN1* ortholog, SHOOT MERISTEMLESS (STM), with AS1 in turn negatively regulating the expression of two other *KNOX1* genes, *AtKNAT1* and *AtKNAT2* [34,38,64]. However, in compound leaf species, the regulatory relationships between the orthologs of these genes are more complex with the expression of *KNOX1* genes generally extending from the SAM to the leaf primordia [36,65]. In tomato for example, *SlTKN1* and *SlTKN2* are expressed throughout the SAM with the expression of both *KNOX1* genes extending to the peripheral zone of the SAM where leaf primordia initiate [66,67,68], and where the *SlPHAN* gene has also been demonstrated to be expressed [36]. Therefore, although we failed to establish interactions at either the genetic or molecular level between *SlDEM1*, *SlPHAN*, *SlTKN1* and *SlTKN2*, the expression analyses presented in Figure 6A indicate that in the tomato hypocotyl, DEM1 positively influences the expression of the *SlPHAN* and *SlTKN1* genes, while negatively regulating the abundance of the *SlTKN2* transcript. Further, the *SlPHAN*, *SlTKN1* and *SlTKN2* expression trends presented in Figure 6A provide additional weight to the theory that the molecular relationships between *PHAN-LIKE* and *KNOX1* genes in compound leaf species such as tomato are quite distinct to the well documented relationships of their gene orthologs in simple leaf species such as *Arabidopsis*.

Considering that tomato encodes a second *DEM* gene, *SlDEM2*, in addition to *SlDEM1*, the extremely high degree of severity of the phenotype displayed by the *dem1* mutant throughout the seedlings stage of vegetative development strongly infers that the *SlDEM1* and *SlDEM2* genes encode functionally distinct proteins. However, protein sequence analysis showed that *SlDEM2* is highly similar to *SlDEM1*, a degree of similarity that strongly infers that the protein products encoded by the two tomato *DEM* genes must have some level of functional overlap. Analysis of the expression of *SlDEM1* and *SlDEM2* revealed contrasting expression profiles for the two tomato *DEM* genes, namely, northern blotting revealed *SlDEM1* expression to be primarily concentrated in the apex, young leaves and floral buds of WT tomato plants, whereas the *SlDEM2* transcript was detected across all assessed tomato tissues with its abundance peaking in the floral buds (Figure 6B). The lack of expression of even a mild phenotype during reproductive development in the *SMD* transformant line could potentially be accounted for by the ability of *SlDEM2* to compensate for disrupted *SlDEM1* gene function in this tissue where both tomato *DEM* genes are expressed. Furthermore, although Figure 6B northern blotting data also clearly shows that *SlDEM1* and *SlDEM2* are both expressed in the apex of WT tomato plants, we have previously shown that the *SlDEM1* transcript accumulates in highly specific regions of this developmentally important tissue [41]. More specifically, in situ hybridization analysis revealed *SlDEM1* transcript accumulation to be restricted to the central zone of the SAM, initiating leaf primordia, axillary meristems, and the adaxial tissues of initiating leaves. This finding when considered together with the severe developmental phenotype expressed by *dem1* mutant seedlings (Figure 1), strongly suggests that although the *SlDEM2* gene is expressed in apical tissues of WT tomato plants, its specific pattern of localized expression in this developmentally important region must differ to the previously and comprehensively documented expression pattern for *SlDEM1*. However, a rigorous experimental approach such as that used previously to characterize *SlDEM1* gene expression in the developing tissues of WT tomato seedlings [41] is required in the future to uncover the exact expression domain of *SlDEM2* in the apex of WT tomato plants to confirm this hypothesis. A shared and/or common function is likely for the *SlDEM1* and *SlDEM2* proteins; however, the function of each tomato DEM protein is likely to be restricted to specialized cell types of each tissue in which the *SlDEM1* and *SlDEM2* genes are co-expressed, as has been demonstrated previously for each member of the YABBY transcription factor family in *Arabidopsis* [69,70]. More specifically, each member of the *Arabidopsis YABBY* gene family shares a common role in the establishment of polarity for each of the different above ground lateral organs, yet each family member only specifies its function in the organs in which it is expressed [69,70]. Thus, in addition to performing in situ hybridization analysis to document the tissue-specific expression of *SlDEM2*, a somatic mutagenesis approach similar to that reported here which was successfully used to assign new roles to the *SlDEM1* protein in tomato vegetative development, should be applied to the tomato *DEM2* gene for the assignment of tissue- or even cell-type specific function to the *SlDEM2* protein.

## 4. Materials and Methods

### 4.1. Plant Growth and Plant Transformation

The commercial cultivar Moneymaker was used for all reported tomato experimentation. All tomato lines described in this study, including the *dem1*, *dem^Ds^* and *dem^+7^* mutant lines, and the *sAc*, *SMD*, *Ex-2*, *NLS-Ex-2* and *DEM1pro:Ex-1* transformant lines, were cultivated in a naturally illuminated glasshouse that was cooled to 28 °C when the internal temperature exceeded this maximum setpoint. 

For the transformation of the Moneymaker cultivar, a modification of the protocols previously described in detail [71,72] was used. In brief, sterilized seeds were germinated on standard plant growth medium (half strength Murashige and Skoog (½ MS) salts) and cultivated for an 11-day period in a growth room at a constant temperature of 25 °C and a 16 h (h) photoperiod under cool white fluorescent lighting. Once the cotyledons had fully expanded, and prior to the emergence of any true leaves, cotyledons were sectioned into approximate 1.0 cm (cm) lengths and immediately transferred to moistened filter paper. The cotyledon sections were overlayed with 2.0 milliliters (mL) of tobacco suspension culture [71,72] as a feeder culture and incubated for 12–16 h in the dark at 25 °C. The cotyledon sections were transferred to a sterile Petri dish and incubated for 5 min (mins) at room temperature in a liquid culture of *Agrobacterium tumefaciens* (strain LBA4404) confirmed to contain each desired plant expression vector. The cotyledon sections were blotted dry on sterile filter paper, transferred to a new Petri dish containing a fresh aliquot of tobacco suspension culture and incubated for 48 h under cool white fluorescent lighting at 25 °C. Any explant material was detached from the cotyledon sections and transferred to new sterile Petri dishes containing ‘shooting medium’ [71,72] with the appropriate selection (50 μg/mL kanamycin; 300 mg/L Timentin^®^). Subculturing of the explant material onto fresh shooting medium was conducted every 2–3 weeks until healthy shoot material could be collected. Shoots with a height of greater than 5 mm (mm) were excised from the explant material and transferred to sterile magenta boxes containing freshly prepared ‘rooting medium’ [71,72] with the appropriate selection (50 μg/mL kanamycin; 600 mg/L Timentin^®^). Subculturing of the plantlets onto fresh aliquots of rooting medium was continued every 2–3 weeks until the shoot and root system of each plantlet was well established, at which time, such plantlets were transferred to sterilized soil (University of California mix) and cultivated under the growth regime outlined above for WT tomato plants.

The Columbia-0 (Col-0) ecotype was used for all *Arabidopsis* analyses reported here. Col-0 seeds were surface sterilized using 70% (*v*/*v*) ethanol and 2.6% (*v*/*v*) commercial bleach, prior to being collected in 0.15% (*w*/*v*) agar and pipetting directly onto the surface of sterilized soil (University of California mix) in 4.0 cm square pots. The seeds in each pot were vernalized at 4 °C in the dark for 4 days, and then the pots were transferred to an *Arabidopsis* growth room with a constant temperature of 21 °C and a 16 h photoperiod under cool white fluorescent lighting.

The floral dip transformation method described previously by [73] was used together with *Agrobacterium* strain GV3101 to introduce the *DEM1pro:Ex-1* or *DEM1pro:DEM1-FL-CDS* transgenes into *Arabidopsis* Col-0 plants. In brief, the floral material of 6-week-old Col-0 plants was removed to only leave the terminal floral bud of the primary inflorescence. The prepared Col-0 plants were inverted and swirled gently by hand for 20–30 s (s) in 50 mL of dipping media (*Agrobacterium* containing the desired plasmid-based plant expression vector; 5.0% sucrose (*w*/*v*); 0.375% Sliwet L-77 (*v*/*v*)). Dipped Col-0 plants were wrapped loosely in clear plastic film and incubated at room temperature for 24 h in low light. The dipped plants were returned to the *Arabidopsis* growth room and cultivated under the standard growth regime outlined above until fully mature seeds could be harvested. Putative transformants were selected either via cultivation of dipped seeds on *Arabidopsis* growth medium (½ MS medium) containing the appropriate selection (50 μg/mL kanamycin) or via planting the dipped seeds directly onto sterilized soil (University of California mix) and spraying of 2-week-old seedlings with 0.04% (*v*/*v*) Basta^®^ (Crop Solutions Australia, BASF, Melbourne, Australia).

### 4.2. Microscopy Techniques for Tomato Plant Line Analysis

For samples to be prepared for scanning electron microscopy (SEM), the sectioned tissue was fixed twice in formaldehyde acetic acid at 37 °C for 30 min, and then subsequently dehydrated at 67 °C in 70% (*v*/*v*) ethanol for 75 s, 100% (*v*/*v*) ethanol for 75 s, and 100% (*v*/*v*) isopropanol for 90 s at 75 °C. Following the alcohol dehydration series, sections were dried in a critical point dryer, sputter coated to a depth of 20 nanometers (nm) with palladium and viewed on a DS130 scanning electron microscope (ISI, Philadelphia, PA, USA).

To prepare samples for analysis via light microscopy, samples were prefixed with 3.0% (*v*/*v*) glutaraldehyde in 0.1 M cacodylate buffer (Na(CH_3_)2AsO_2_) for 2 h at 4 °C. Each sample was washed three times for 10 min per wash in fresh changes of 0.1 M cacodylate buffer. Post washing, samples were fixed in 0.1 M cacodylate buffer containing 1.0% (*w*/*v*) osmium tetroxide for 2 h at 4 °C. The fixed samples were again washed with three changes of fresh 0.1 M cacodylate buffer which was then followed by two 10 min washes with fresh changes of water. Post washing, samples were dehydrated through a graded acetone series and were next infiltrated using Spurr’s resin with the resin being polymerized by incubation of the samples at 65 °C for 72 h. The prepared samples were processed into 20 μm sections with an Ultracut E microtome (Reichert-Jung, Buffalo, MD, USA), and the resulting sections were stained in toluidine blue and examined under a light microscope.

### 4.3. Plant Expression Vector Construction and Introduction into Agrobacterium tumefaciens

The bacterial cloning steps involved in the construction of the *sAc* and *Ds* plant expression vectors prior to their introduction into wild-type tomato (cv., Moneymaker) plants has been described in detail previously [41,43,74]. Components of the previously described plasmid vectors, pSLJ4K1 [75], pSLJ81 [75] and pBI121 [76] were used in the construction of the *Ex-2*, *NLS-Ex-2*, *DEM1pro:Ex-1* and *DEM1pro:DEM1-FL-CDS* plant expression vectors developed for use in this study. In brief, to construct the *Ex-2* plant expression vector, primers *Ex2-F* and *Ex2-R* were used to amplify the *DEM1 Ex-2* sequence from tomato genomic DNA and the resulting amplicon was introduced into vector pSLJ4K1 [75] via *Cla*I and *Xba*I restriction endonuclease digestion and a standard bacterial cloning approach to produce vector pSLJ4K1:*Ex-2*. The *Ds* element was subsequently introduced into the pSLJ4K1:*Ex-2* vector via digestion of vectors pSLJ4K1:*Ex-2* and pSLJ81 [75] with *Sal*I and *Sac*I and ligation of the resulting restriction fragments. This approach allowed for the generation of the *35Spro*:*Ds*:*DEM1 Ex-2*:*nosT* transgene which was subsequently introduced into tomato plants (as outlined below) to produce *Ex-2* transformants. To generate the *NLS-Ex-2* plant expression vector, primers *NLS-F* and *NLS-R* were used to amplify the putative *NLS* encoded by *DEM1 Ex-1*, together with the *DEM1 Ex-2* coding sequence, from a full-length *SlDEM1* cDNA. The resulting amplicon, and the vector pSLJ4K1:*Ex-2*, were next digested with *Cla*I and *Xba*I to replace the existing *DEM1 Ex-2* fragment of the pSLJ4K1:*Ex-2* vector with the *NLS-Ex-2* sequence. The *Ds* element was subsequently introduced into the resulting pSLJ4K1:*NLS-Ex-2* vector as outlined above for the *35Spro*:*Ds*:*DEM1 Ex-2*:*nosT* transgene to produce the *35Spro*:*Ds*:*NLS-DEM1 Ex-2*:*nosT* transgene, which was subsequently introduced into tomato plants (as outlined below) to produce *NLS-Ex-2* transformants.

To construct plant expression vector, *DEM1pro:Ex-1*, primers *DEM1PRO-F* and *Ex1-R* were used in a standard PCR approach to amplify the *DEM1* promoter and the first exon of the gene as a single amplicon from tomato genomic DNA. Following digestion with restriction endonucleases, *Bcl*I and *Pst*I, the *DEM1pro*:*Ex-1* PCR product and the pSLJ4K1 vector were ligated together to produce vector pSLJ4K1:*DEM1pro*:*Ex-1*. In a separate PCR, primers *3UTR-F* and *3UTR-R* were used to amplify the *DEM1* 3′ UTR with the resulting amplicon digested with *Xba*I to allow for its introduction into the *Xba*I digested pSLJ4K1:*DEM1pro*:*Ex-1* vector immediately downstream of the existing *DEM1pro*:*Ex-1* insert. The resulting *DEM1pro*:*Ex-1*:*3′ UTR*:*nosT* transgene was then used to transform either tomato or *Arabidopsis* plants via the respective approaches outlined here for these two species. The *DEM1 Ex-1* sequence of the pSLJ4K1:*DEM1pro*:*Ex-1*:*3UTR* vector was replaced with a PCR generated fragment representing the full-length CDS of the *SlDEM1* gene following digestion of the vector and amplicon with *Bam*HI and *Xba*I and ligation of the resulting restriction fragments. The resulting *DEM1pro*:*DEM1-FL-CDS*:*3′ UTR*:*nosT* transgene was then introduced into tomato and *Arabidopsis* via the *Agrobacterium*-mediated transformation approaches outlined here to generate tomato and *Arabidopsis DEMpro*:*DEM1-FL-CDS* transformants.

Post plant expression vector construction, a triparental mating approach was used to introduce each binary vector into *Agrobacterium* (strain LBA4404). Specifically, bacterial cells were pelleted via centrifugation at 7,000 rpm for 10 min at 4 °C from 10 mL liquid Luria-Bertani (LB) medium cultures of (1) *Agrobacterium* LBA4404 (cultured at 28 °C for 36 h with shaking), (2) *Escherichia coli* (*E. coli* strain DH5α) harboring each plasmid-based plant expression vector (cultured at 37 °C for 12 h with shaking), and (3) *E. coli* DH5α containing the helper plasmid, pRK2013 [77], which had been cultured at 37 °C for 12 h with shaking. Each pelleted cell preparation was resuspended in 1.0 mL of liquid LB medium via careful pipetting and then 30 μL of each bacterial resuspension was plated out on to a non-selective plate of solid LB medium and incubated at 28 °C for 16 h. Bacterial colonies which had formed were used to streak selective (50 μg/mL rifampicin; 50 μg/mL kanamycin) plates of solid LB medium with the plates incubated at 28 °C for 48 h. Single bacterial colonies were then used to streak an additional selective (50 μg/mL rifampicin; 50 μg/mL kanamycin; 25 μg/mL streptomycin) plate of solid LB medium which was incubated at 28 °C for 48 h. Single colonies were then screened via PCR to confirm (1) the presence of each introduced plant expression vector, and (2) that conjugation had been successful.

### 4.4. Genomic DNA Extraction and Nucleic Acid Hybridization

High quality genomic DNA was extracted from young leaves or the apices of each assessed tomato transformant line using the method previously described in detail [43]. In brief, 20 μg of genomic DNA was digested for 16 h at 37 °C with 100 units (U) of the appropriate restriction endonuclease according to the manufacturer’s instructions (New England BioLabs, Melbourne, Australia). Post purification of the digested genomic DNA, 15 μg of digested genomic DNA was separated on a 0.7% (*w*/*v*) agarose gel via electrophoresis. Capillary blotting was used to transfer the digested genomic DNA onto a positively charged HyBond-N^+^ nylon membrane (Sigma Aldrich, Sydney, Australia) with the transferred DNA subsequently fixed to the membrane via crosslinking in a Stratagene UV CrossLinker 2400 (Stratagene, San Diego, CA, USA) at 450 millijoules (mJ). Membranes were prehybridized at 65 °C for 12 h in 25 mL of hybridization buffer which contained 300 μg of denatured salmon sperm DNA in 0.5 M Na_2_HPO_4_ (pH 7.2), 7.0% SDS (*w*/*v*) and 10 mM EDTA. Each DNA probe was labeled with α^32^P-dCTP using the MegaPrime™ Labeling System according to the manufacturer’s protocol (Sigma Aldrich, Sydney, Australia), and post labeling, unincorporated nucleotides were removed from the labeled probe using a MicroSpin™ S-400 HR column (Sigma Aldrich, Sydney, Australia). The labeled, purified probe was denatured via heating and then incubated with the prehybridized membrane for 16 h at 65 °C in a hybridization oven with constant rotation. Probed membranes were washed with a series of buffers of increasing stringency exactly as outlined in [43]. Post washing, membranes were sealed in plastic envelops and exposed to phosphor screens for 16–24 h for data visualization using a PhosphorImaginer. The sequences of the DNA oligonucleotides used as primers to generate the *NPTII* and *BAR* amplicons by PCR for use as probes for Southern blot hybridization analysis are listed in Supplementary Table S1.

### 4.5. Total RNA Extraction, Complementary DNA Synthesis, and Reverse-Transcriptase Polymerase Chain Reaction

For total RNA extraction, 100 mg of plant material was sampled from the desired organ, tissue type or stage of tomato development, and immediately frozen in liquid nitrogen (LN_2_). Each plant tissue sample was ground into a fine powder under LN_2_ using a LN_2_ cooled mortar and pestle, then the ground tissue powder was immediately transferred to a LN_2_ cooled 1.5 mL microfuge tube. One milliliter of TRIzol™ Reagent was used for all total RNA extractions, exactly as outlined in the manufacturer’s protocol for plant tissue samples (Thermo Fisher Scientific, Brisbane, Australia). The quality of each total RNA extraction was determined via electrophoresis on an ethidium bromide (EtBr)-stained 1.2% (*w*/*v*) agarose gel and visualization on a UV illuminator. The concentration of each total RNA extraction was determined via the use of a GeneQuant spectrophotometer (Pharmacia, Rockville, MD, USA). For each total RNA extraction deemed to be of acceptable quality, 20 μg of sample was electrophoresed on a 1.2% (*w*/*v*) agarose gel that contained formaldehyde for 4 h at 40 volts (V) at room temperature. The denatured and separated total RNA was subsequently transferred to HyBond-N^+^ nylon membranes, fixed to the positively charged membranes by UV-crosslinking, pre-hybridized, hybridized, and visualized via the use of Phosphor screens exactly as outlined above for the Southern blot hybridization approach. The sequences of the DNA oligonucleotides used as primers to generate full length *SlDEM1*, *SlDEM2*, *SlPHAN*, *SlTKN1* and *SlTKN2* amplicons by PCR for use as probes for northern blot hybridization analysis are listed in Supplementary Table S1. 

The Superscript (SuperScript II Reverse Transcriptase) First Strand Synthesis System was used to synthesize first strand complementary DNA (cDNA) from 1.0 μg of DNase I-treated total RNA exactly as outlined by the manufacturer (Thermo Fisher Scientific, Brisbane, Australia). The use of quantitative reverse transcriptase PCR (RT-qPCR) to determine the degree of overexpression of exon-2 of *SlDEM1* in the *Ex-2* and *NLS-Ex-2* transformant lines was conducted using the cycling conditions; (1) 1 × 95 °C for 10 min, and (2) 45 × 95 °C for 10 s and 60 °C for 15 s, and with the GoTaq^®^ qPCR Master Mix (Promega, Sydney, Australia) used as the fluorescent reagent. The sequences of the DNA oligonucleotides used as primers for the RT-qPCR assessments are listed in Supplementary Table S1.

## 5. Conclusions

Here, we show that in tomato, loss of *DEM1* gene function results in seedling lethality with the mutant phenotype displayed by *dem1* seedlings characterized by a (1) highly disorganized SAM, (2) failure of leaf primordia to initiate, (3) reduced size and highly variable shape of cotyledon adaxial epidermis cells, (4) lack of palisade mesophyll cell organization and the formation of palisade mesophyll cells of reduced size and variable shape, (5) enhancement of the proliferation of spongy mesophyll cells, and (6) normal program of development of the abaxial epidermis of the cotyledons and all cell and tissue types of the hypocotyl. The *SMD* transformant line allowed for the assignment of additional roles to the *DEM1* gene post the seedling stage of tomato vegetative development. Specifically, the developmental phenotypes displayed by *SMD* leaves allowed for the assignment of *SlDEM1* gene function to the (1) control of leaf blade lateral expansion, (2) regulation of the size and shape of adaxial epidermal cells, (3) control of adaxial trichome development, (4) promotion of palisade mesophyll cell proliferation, and (5) repression of spongy mesophyll cell proliferation. Furthermore, the sectored or stable overexpression of the second exon of *SlDEM1* with or without additional regulatory sequences also derived from the *SlDEM1* coding sequence confirmed the absolute requirement of *SlDEM1* gene function for normal leaf adaxial epidermis cell differentiation and mesophyll cell proliferation. In addition, the stable expression of the first exon of the tomato *DEM1* gene improved embryo fitness yet failed to fully complement the seedling lethality of the *dem1* mutant: a finding which further highlighted the central role occupied by the *DEM1* gene in the early stages of embryo development in tomato. In addition to *DEM1*, the tomato genome encodes a second *DEM* gene, *SlDEM2*, with our analyses revealing *SlDEM1* and *SlDEM2* expression to overlap in developmentally important tissues such as the shoot apex and floral buds. However, although the two tomato *DEM* genes encode highly similar proteins, the seedling lethality of the *dem1* mutant strongly suggests that the two *SlDEM* genes perform their yet to be determined biochemical function in highly specific cell types and stages of tomato development.

## Data Availability

All plant material reported in this study can be requested from the corresponding author B.J.C upon request.

## Supplementary Materials

The following supporting information can be downloaded at: https://www.mdpi.com/article/10.3390/plants11192545/s1. Table S1: DNA oligonucleotides used in this study.
