# Supplementary material for: DEFECTIVE EMBRYO AND MERISTEMS1 (DEM1) Is Essential for Cell Proliferation and Cell Differentiation in Tomato
Source: Plants (Basel). 2022 Sep 28;11(19):2545. doi: 10.3390/plants11192545 (PMC9573268; doi:10.3390/plants11192545)
Supplement: Supplementary file 1 [file plants-11-02545-s001.zip › plants-1932510-supplementary.pdf]

DEFECTIVE EMBRYO AND MERISTEMS1 (*DEM1*) Is Essential for Cell Proliferation and Cell Differentiation in Tomato

Louisa Matthew [1,†], Melquiades E. C. Reyes [1,†], Christopher W. G. Mann [1], Alasdair W. McDowall [2,3], Andrew L. Eamens [1,4,*,†] and Bernard J. Carroll [1,*,†]

[1] School of Chemistry and Molecular Biosciences, The University of Queensland, St. Lucia, QLD 4072, Australia
[2] Centre for Microscopy and Microanalysis, The University of Queensland, St. Lucia, QLD 4072, Australia
[3] California Institute of Technology, Pasadena, CA 91125, USA
[4] School of Health and Behavioural Sciences, University of the Sunshine Coast, Maroochydore, QLD 4558, Australia
* Correspondence: aeamens@usc.edu.au (A.L.E.); b.carroll@uq.edu.au (B.J.C.)
† These authors contributed equally to this work.

Table S1. DNA oligonucleotides used in this study. The 5′ to 3′ sequence of each DNA oligonucleotide used in this study for plant expression vector construction, *SMD* transformant analysis, RT-qPCR analysis, and Southern and northern blot hybridization analysis is listed below with the specific use of each DNA oligonucleotide provided in the Materials and Methods section.

Oligonucleotide Name	Target Sequence / Oligonucleotide Use	Sequence (5′ – 3′)
**Plant Expression Vector Construction**		
DEM1-PRO-F	*SlDEM1* promoter	GGGTGATCACTGCAGTAATTTATATTGACATAATGAAGGC
Ex1-R	*SlDEM1* exon-1	CCCTCTAGACCCATGAAAATTGAGCACTGCGGAACTTG
3UTR-F	*SlDEM1* 3′ UTR	CCCTCTAGAACAATCATTCTGTTGATATATGCAACTTATTAG
3UTR-R	*SlDEM1* 3′ UTR	AGACTACTTGCAGTTAAGAAGGAGATAGAGGCT
Ex2-F	*SlDEM1* exon-2	GGGATCGATGGTCACCGAGAATGGGAAGCAAGAG
Ex2-F	*SlDEM1* exon-2	CCCTCTAGATCATATTTGTAAGCGCCTGCTAGAG
NLS-F	*SlDEM1* NLS	GGGATCGATGGTTAACCATGATTTGAGGGAGGAGTTTGAGGAGGCA
**SMD Transformant Analysis (mapping of *Ds* insertions into the *SlDEM1* locus)**		
DEM5	*SlDEM1* 5′ terminus	TTTCTCCTCCTTAAATGCATTGAG
DEM3	*SlDEM1* 3′ terminus	TTCATGTTGGTGGGAACACTGCGA
B34	*Ds* element (3′ region)	ACGGTCGGTACGGGATTTTCCCAT
B39	*Ds* element (5′ region)	GTTTTCGTTTCCGTCCCGCAA
D71	*Ds* element (5′ region)	CCGTTACCGACCGTTTTCATCCCTA
D73	*Ds* element (3′ region)	GAACGCGACATAATGGTTTCTGACG
**Reverse Transcriptase Quantitative Polymerase Chain Reaction (RT-qPCR) Analysis**		
TUB-RTF	*SlTUB1* gene	CGATTCCGTTCTTGT
TUB-RTR	*SlTUB1* gene	AATGAGTGACACACTTGGAATCCTT
Ex2-RTF	*SlDEM1* exon-2	CGCTGTTTCTGACTCACCTGAA
Ex2-RTR	*SlDEM1* exon-2	GCCTGCTAGAGATGCTGAATGA
**Probe Synthesis For Nucleic Acid Hybridization (Southern or Northern Blotting) Analysis**		
OLI-DT15	mRNA poly-A tails	TTTTTTTTTTTTTTT
DEM1-PF	*SlDEM1* cDNA	TTCAAATGGGTGCTAATCACAGC
DEM1-PR	*SlDEM1* cDNA	ATTGTTCAAATTTGTAAGCGCCT
DEM2-PF	*SlDEM2* cDNA	GAGAAATGTCCACCATGGAACTCGTTG
DEM2-PR	*SlDEM2* cDNA	CCCTTTGTTTACGGATAAAGCTGGGAAG
DEM2-PF2	*SlDEM2* cDNA	CCCAGCTATATGCGCATCCACAGGAGTCAG
DEM2-PR2	*SlDEM2* cDNA	GGATTAAAGCTGACTCCTGTGGATGCG
PHAN-PF	*SlPHAN* cDNA	GGGGAATTCATGAGGAGAGGCAACGG
PHAN-PR	*SlPHAN* cDNA	GGGGGAAGTCGCGGCAAGTAGAAA
TKN1-PF1	*SlTKN1* cDNA	CTTGATTTAGGGTTTTTTTTCCCC

TKN1-PR1	*SlTKN1* cDNA	GTGCTTAGGCCTATAAGGCTTGC
TKN1-PF2	*SlTKN1* cDNA	GGGGAATTCATGGAGAATAATAATTATAATAATCATGTGTCTGG
TKN1-PR2	*SlTKN1* cDNA	GGGGGATCCAAAATTCATAAATATTACTGACCCAAACGAAAAGG
TKN2-PF1	*SlTKN2* cDNA	GATCACTTACACACACACACACACCC
TKN2-PR1	*SlTKN2* cDNA	GCTTGTACTACTACATGCACAC
TKN2-PF2	*SlTKN2* cDNA	GGGGAATTCATGGAGGGTGGTTCTAGTGG
TKN2-PR2	*SlTKN2* cDNA	GATCACTTACACACACACACACACCC
BAR-R-PF	*BAR* gene	CGAGATAGGGTTGAGTGTTGTTCC
BAR-R-PR	*BAR* gene	AGACAAGCACGGTCAACTTCCGTA
NPTII-PF	*NPTII* gene	GAATTAAGGGAGTCACGTTATGACC
NPTII-PR	*NTPII* gene	CAGAAGAACTCGTCAAGAAGGCG
